# Synthesis and Polyelectrolyte Functionalization of Hollow Fiber Membranes Formed by Solvent Transfer Induced Phase Separation

**DOI:** 10.1021/acsami.2c10343

**Published:** 2022-09-15

**Authors:** Henrik Siegel, Alessio J. Sprockel, Matthew S. Schwenger, Jesse M. Steenhoff, Iske Achterhuis, Wiebe M. de Vos, Martin F. Haase

**Affiliations:** † Van’t Hoff Laboratory of Physical and Colloid Chemistry, Department of Chemistry, Debye Institute for Nanomaterials Science, Utrecht University, 3584 CH Utrecht, The Netherlands; ‡ Henry M. Rowan College of Engineering, Rowan University, Glassboro, New Jersey 08028, United States; § Faculty of Science and Technology, Membrane Surface Science, Membrane Science and Technology, MESA+ Institute of Nanotechnology, University of Twente, 7500 AE Enschede, The Netherlands

**Keywords:** nanoparticles, self-assembly, separation membranes, polyelectrolytes, ultrafiltration

## Abstract

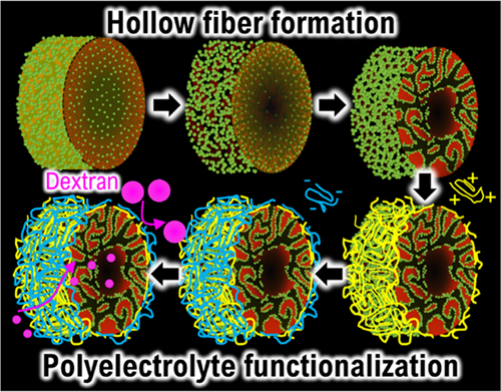

Ultrafiltration membranes are important porous materials to produce freshwater in an increasingly water-scarce world. A recent approach to generate porous membranes is solvent transfer induced phase separation (STrIPS). During STrIPS, the interplay of liquid–liquid phase separation and nanoparticle self-assembly results in hollow fibers with small surface pores, ideal structures for applications as filtration membranes. However, the underlying mechanisms of the membrane formation are still poorly understood, limiting the control over structure and properties. To address this knowledge gap, we study the nonequilibrium dynamics of hollow fiber structure evolution. Confocal microscopy reveals the distribution of nanoparticles and monomers during STrIPS. Diffusion simulations are combined with measurements of the interfacial elasticity to investigate the effect of the solvent concentration on nanoparticle stabilization. Furthermore, we demonstrate the separation performance of the membrane during ultrafiltration. To this end, polyelectrolyte multilayers are deposited on the membrane, leading to tunable pores that enable the removal of dextran molecules of different molecular weights (>360 kDa, >60 kDa, >18 kDa) from a feed water stream. The resulting understanding of STrIPS and the simplicity of the synthesis process open avenues to design novel membranes for advanced separation applications.

## 1 Introduction

Synthetic membranes enable the separation of mixtures with relatively low energy requirements, gentle process conditions, and scalable throughputs.^1,2^ The increasing global water demand and the growing contamination of natural freshwater resources call for advances of membrane materials and synthesis techniques.^3,4^ Conventional membranes are fabricated by precipitation of porous polymer scaffolds via non-solvent induced phase separation (NIPS) with controllable permeability, selectivity, and mechanical strength.^5−7^ Nanocomposite membranes combine the features of polymer membranes with the complementary properties of nanoparticle additives.^8,9^ Nanoparticle inclusions can, for instance, provide antifouling, antimicrobial, catalytic, and adsorptive functionalities to the membrane.^10−13^

Nanocomposite membranes can be synthesized via NIPS by the direct addition of nanoparticles to the NIPS precursor solution, or via postprocessing of the NIPS membrane. However, the uniform distribution and amount of nanoparticles in the NIPS membranes are limited.^14,15^ Solvent transfer induced phase separation (STrIPS) is a novel approach to generate nanocomposite membranes with high loadings of nanoparticles within the membrane (up to 50 wt %).^16−18^ STrIPS nanocomposite membranes are formed by liquid–liquid phase separation and polymerization of precursor dispersions containing tens of weight percentages of well-dispersed nanoparticles.^19−22^ The tensile strengths (100 kPa–15 MPa) and elastic moduli (100 kPa–1 GPa) of STrIPS membranes can be controlled via postprocessing.^23,24^ Moreover, compared to NIPS, STrIPS results in membranes made of cross-linked polyacrylates, facilitating their direct use in organic solvent filtration.^16,18^ Similarly, the cross-linked STrIPS membranes can potentially withstand aggressive cleaning treatments involving acidic, alkaline, chlorine, organic, or oxidizing solutions. STrIPS fiber membranes also evolve hollow interiors by phase separation, simplifying the fiber spinning equipment for the synthesis of hollow fiber membranes. However, the mechanisms producing the hollow interior of STrIPS fibers are not fully understood yet, limiting the control over membrane structure. Here, we investigate the processes behind STrIPS hollow fiber formation to address this knowledge gap.

Furthermore, we tailor the STrIPS hollow fiber membrane selectivity by functionalizing the nanoparticle covered membrane surface with polyelectrolytes. Previously, STrIPS membranes have been used for filtration of gold particles (20 nm) from water.^16^ The separation has been explained based on the sieving of the gold nanoparticles by the membrane surface pores. Nevertheless, the surface pores of STrIPS membranes are not small enough to also separate dissolved lower molecular mass compounds. Membrane surface modifications are needed to enhance ultrafiltration performance with STrIPS templated membranes. A well-established approach to tune the selectivity of porous filtration support membranes is the surface modification with polyelectrolytes.^25−29^ Polyelectrolyte multilayers (PEMs) provide additional control over the membrane separation properties by the removal of lower molecular weight compounds from feed streams.^30^

In this work, we show that PEM functionalization of STrIPS membranes enables aqueous ultrafiltration of dextran with molecular weight cutoffs (MWCO) scaling from 360 to 60 kDa and 18 kDa depending on the thickness of the PEM. This demonstrates the tunability of STrIPS membrane selectivity and exemplifies the versatility of possible surface functionalizations for STrIPS membranes. We begin our manuscript with a discussion of the structure formation mechanisms of the hollow fiber membrane formed via STrIPS. To this end, confocal microscopy, pendant drop tensiometry, numerical diffusion simulations, and ζ-potential measurements are combined to analyze the dynamics of the membrane evolution driven by phase separation and nanoparticle stabilization. By varying the composition of the precursor dispersion, control over the fiber structure is demonstrated. We form a surfactant–polyelectrolyte complex around the STrIPS hollow fibers followed by the deposition of PEMs to transform them into ultrafiltration membranes with tunable pore size. The STrIPS membrane separation performance is characterized via pure water permeability and MWCO.

## 2 Results and Discussion

### 2.1 Structure Control via STrIPS

STrIPS nanocomposite membranes are generated with a homogeneous precursor solution containing well-dispersed silica nanoparticles (Ludox TMA). The liquid precursor dispersion is composed of butanediol diacrylate (BDA), ethanol, water, Ludox TMA nanoparticles, 2-hydroxy-2-methylpropiophenone (HMPP), and hexadecyltrimethylammonium cations (CTA^+^) (for exact compositions, see the Experimental Section). BDA and water are immiscible, but with sufficient ethanol as a solvent, a clear solution can be obtained. To determine the required ethanol volume fractions (φ_EtOH_) for mixing BDA with water, we measure the ternary liquid phase diagram via turbidimetry and confocal microscopy analysis (Figure 2a, see Supporting Information (SI) Section S1).

**Figure 1:**
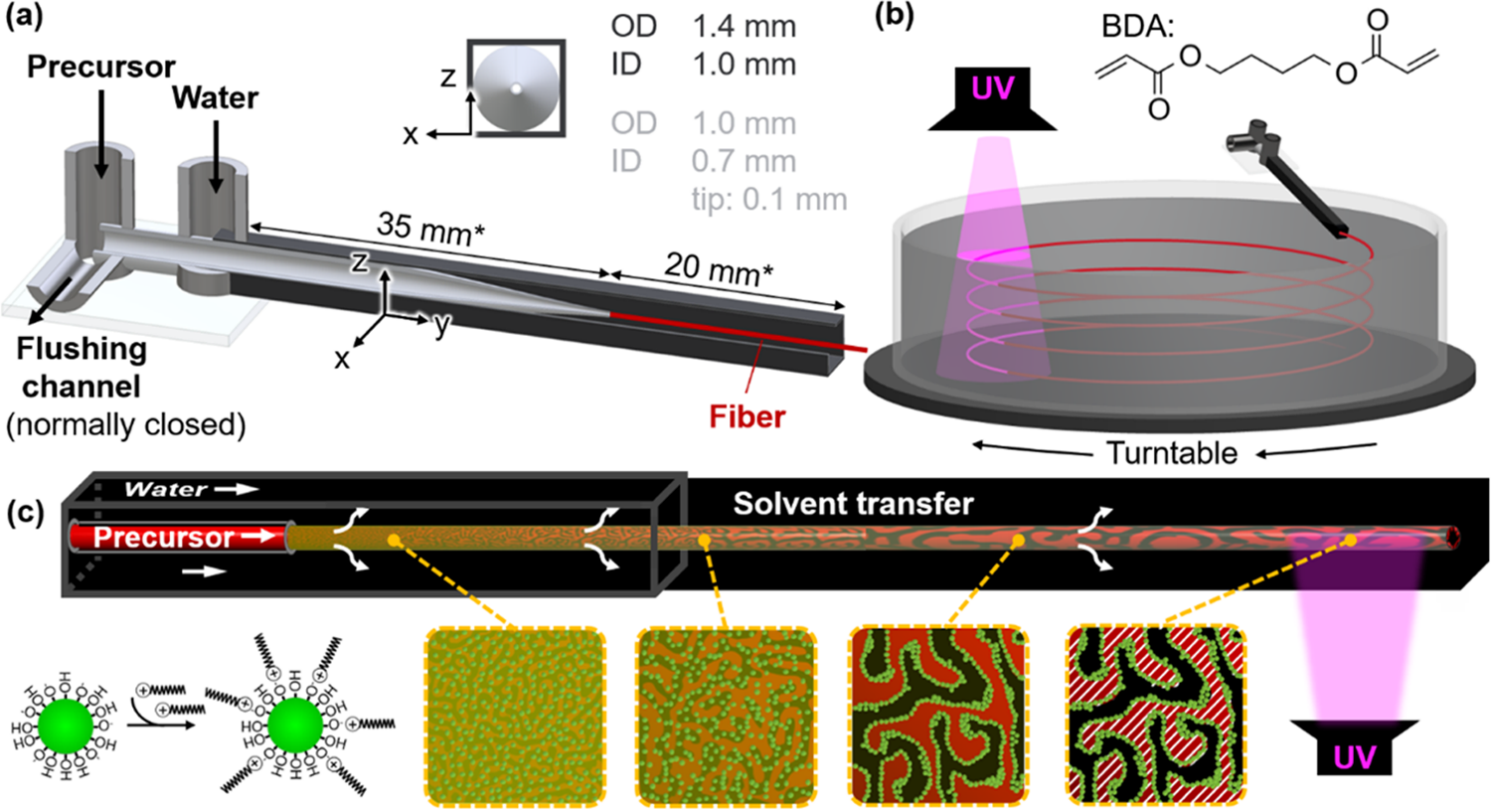
Fiber fabrication via STrIPS. (a) Cutaway drawing, showing the inside of the fiber extrusion device with a tapered inner round capillary (light gray) aligned in a larger square capillary (dark gray). Capillary dimensions are given as outer diameter (OD) and inner diameter (ID), dimensions marked by asterisk (*) are not drawn to scale. (b) Fiber spinning into a rotating water bath and fiber rigidification by UV-light-initiated polymerization of butanediol diacrylate (BDA). (c) Schematic depictions of the CTA^+^ functionalization of the silica nanoparticles and the fiber structure evolution during STrIPS. The nanoparticles assemble at the BDA/water interface formed by phase separation. Black color represents water, red color BDA, red/white dashed regions polyBDA, and green color silica nanoparticles.

A microfluidic device made of glass capillaries is employed to initiate STrIPS. The liquid precursor dispersion is pumped out of the 100 μm orifice of a tapered, round cross-section capillary. From there, it enters into a square cross-section capillary, carrying a co-flowing stream of water with 5 vol % ethanol (Figure 1a, see SI Section S3 for details about microfluidic device assembly).^16,18^ Once the precursor dispersion contacts the water stream, it turns into a viscoelastic fiber due to the diffusion of ethanol. The fiber exits the microfluidic device into a rotating water reservoir supported on a turntable (Figure 1b). In this rotating container, the fibers sinks on a winding path down to the bottom, enabling the collection of circularly arranged fiber segments.

The diffusion of ethanol from the precursor dispersion to the water causes BDA and water to phase separate. The phase separation generates an interface between the BDA- and water-rich phases. Ludox TMA nanoparticles deposit and stabilize this interface. The interfacial deposition of particles is facilitated by their surface functionalization with CTA^+^ molecules. CTA^+^ electrostatically adsorbs on the particles, resulting in a partially hydrophobic surface (Figure 1c). This in turn renders the particles interfacially active. As the phase separation progresses, the particles form a dense layer on the BDA/water interface. Eventually, this nanoparticle layer can arrest the liquid arrangement of the BDA- and water-rich phases via interfacial jamming.^31,32^ As a result, STrIPS generates a viscoelastic fiber made of a silica/CTA^+^-stabilized BDA/water emulsion gel (Figure 1c).

**Figure 2:**
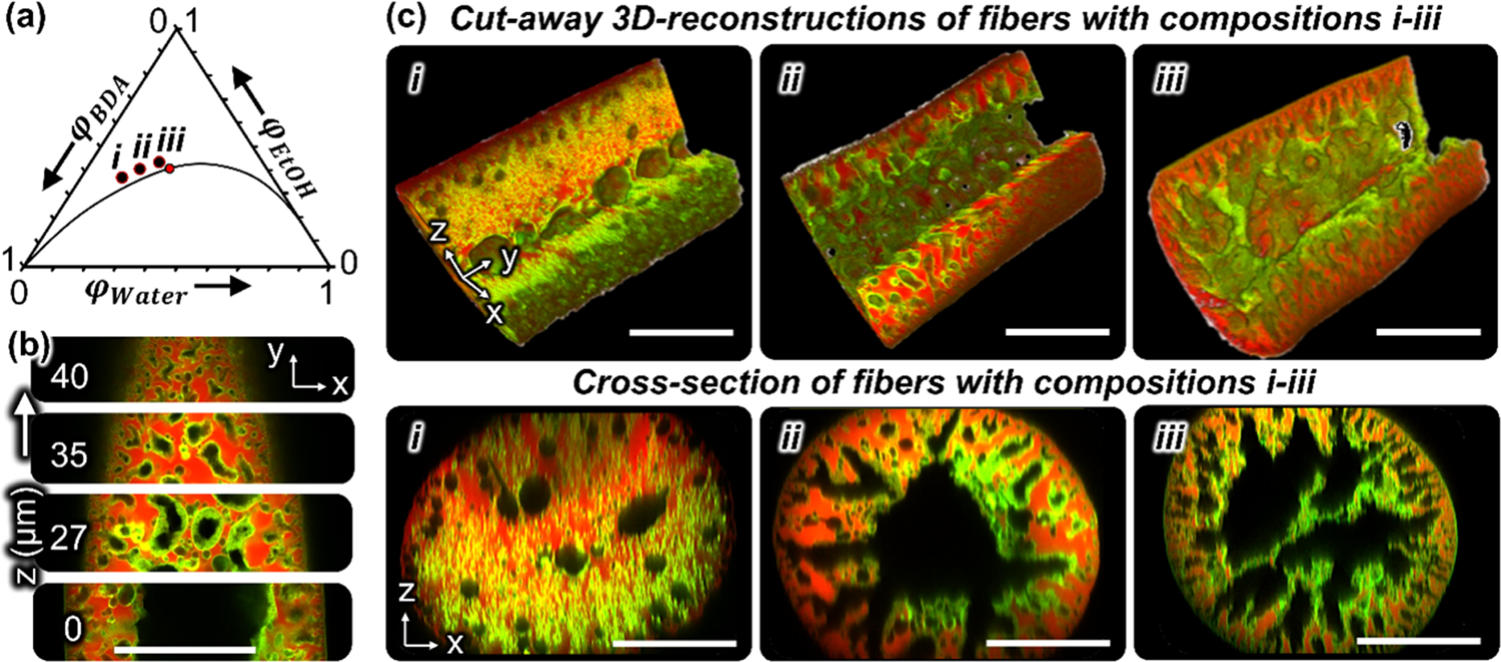
STrIPS fiber structure control. (a) Ternary phase diagram of butanediol diacrylate (BDA), ethanol (EtOH), and water (liquid compositions *i*: φ_BDA_ = 0.433, φ_EtOH_ = 0.399, φ_Water_ = 0.168; *ii*: φ_BDA_ = 0. 367, φ_EtOH_ = 0.418, φ_Water_ = 0.215; *iii*: φ_BDA_ = 0.303, φ_EtOH_ = 0.427, φ_Water_ = 0.270). The red dot on the binodal curve gives the critical point. (b) Confocal microscopy *z*-stack of fiber with composition *ii* represented by micrographs of the fiber acquired from different focal depths *z*. Black color represents water, red color polyBDA, and green color silica nanoparticles. Scale bar 50 μm. (c) Three-dimensional (3D) reconstructions of confocal micrographs of fibers made with the compositions *i*, *ii*, and *iii*. One quarter of the fiber section has been cut away to reveal the 3D fiber interior. Bottom: two-dimensional (2D) cross-sectional views showing more detailed internal structures, nanoparticle and polyBDA distributions. All scale bars are 50 μm.

Confocal microscopy shows the spatial distribution of water, BDA, and nanoparticles within the fiber after STrIPS. To obtain high-quality confocal micrographs, the fiber is photopolymerized, fluorescently labeled with Nile red and made optically transparent. The polymerization of BDA is initiated upon radical generation of HMPP via exposure to a beam of high-intensity UV light (Figure 1b,c). The resulting Ludox TMA/polyBDA composite fiber is washed (see the Experimental Section) and submerged in a 0.1 mol/L NaOH solution of Rhodamine 110 in water. The alkalinity increases the negative charge of the Ludox TMA particles, enabling the adsorption of the positively charged Rhodamine 110. For confocal microscopy, the fibers are placed in diethyl phthalate, a liquid with a refractive index close to polyBDA. With blue laser excitation (488 nm), the Rhodamine 110-stained Ludox TMA particles emit green fluorescence, while the polyBDA domains have red fluorescence from the embedded Nile red (Figure S7). Figure 2b provides a confocal microscopy *z*-stack of a fiber with composition *ii* in Figure 2a. The polyBDA and water domains show an interwoven arrangement near the fiber surface with a fluorescent film of nanoparticles at the interface. The green fluorescence signal at the BDA/water interface confirms that the phase separation of BDA and water was stabilized by the CTA^+^-modified Ludox TMA particles.

The fiber structure depends on the liquid starting composition of the precursor dispersion. Figure 2c shows 3D reconstructions of confocal microscopy *z*-stacks of fibers with the initial compositions *i*–*iii* given in Figure 2a. The isometric perspectives of the fibers are shown as cutaway 3D reconstructions, revealing the interior of the fiber. Cross-sectional views of the fibers are provided below the 3D reconstructions in Figure 2c. The micrographs showing short segments are representative for the entire length of the fibers, as well as for fibers prepared with different samples of the same compositions (see SI Section S4). Precursor composition *i* shows isolated water cavities within the fiber. The water cavities suggest that the phase separation has proceeded via nucleation and growth of water droplets in BDA. In contrast, fibers fabricated with compositions *ii* and *iii* show an interwoven arrangement of the polyBDA and water channels. This structure indicates that phase separation has proceeded via spinodal decomposition. The proximity of compositions *iiand* iii to the critical point of the phase diagram in Figure 2a (given as a red dot on the binodal curve) supports this interpretation.

Fibers with compositions *ii* and *iii* have an increasing pore size from the surface toward the interior. The outer pores of 1–2 μm grow to several tens of micrometers toward the center of the fiber, where they merge to a hollow interior. The asymmetric structure suggests that the nanoparticles have successfully stabilized the phase-separated structures near the surface of the fiber but have failed to stabilize the fiber center. The center of these fibers are composed of a large water cavity, which extends along the entire length of the fiber. In Section 2.3, we will use this hollow interior as a permeate drainage channel for membrane filtration. In Section 2.2, we investigate the reason for the inability of the particles to stabilize the fiber interior during phase separation. We therefore first analyze the ethanol diffusion dynamics during STrIPS, and second, the role of the particle surface functionalization with CTA^+^.

### 2.2 Structure Formation Mechanisms

To understand the dynamics of STrIPS, a COMSOL diffusion simulation is employed, calculating the ethanol concentration profiles in and around the fiber over time. The model represents an approximation of the real mass transfer during STrIPS. It considers the undisturbed ethanol diffusion process but does not take the demixing phenomena within the fiber into account (see SI Section S6 for details). Nevertheless, previous work has shown that the model accurately predicts the fiber density evolution over time.^23^

**Figure 3:**
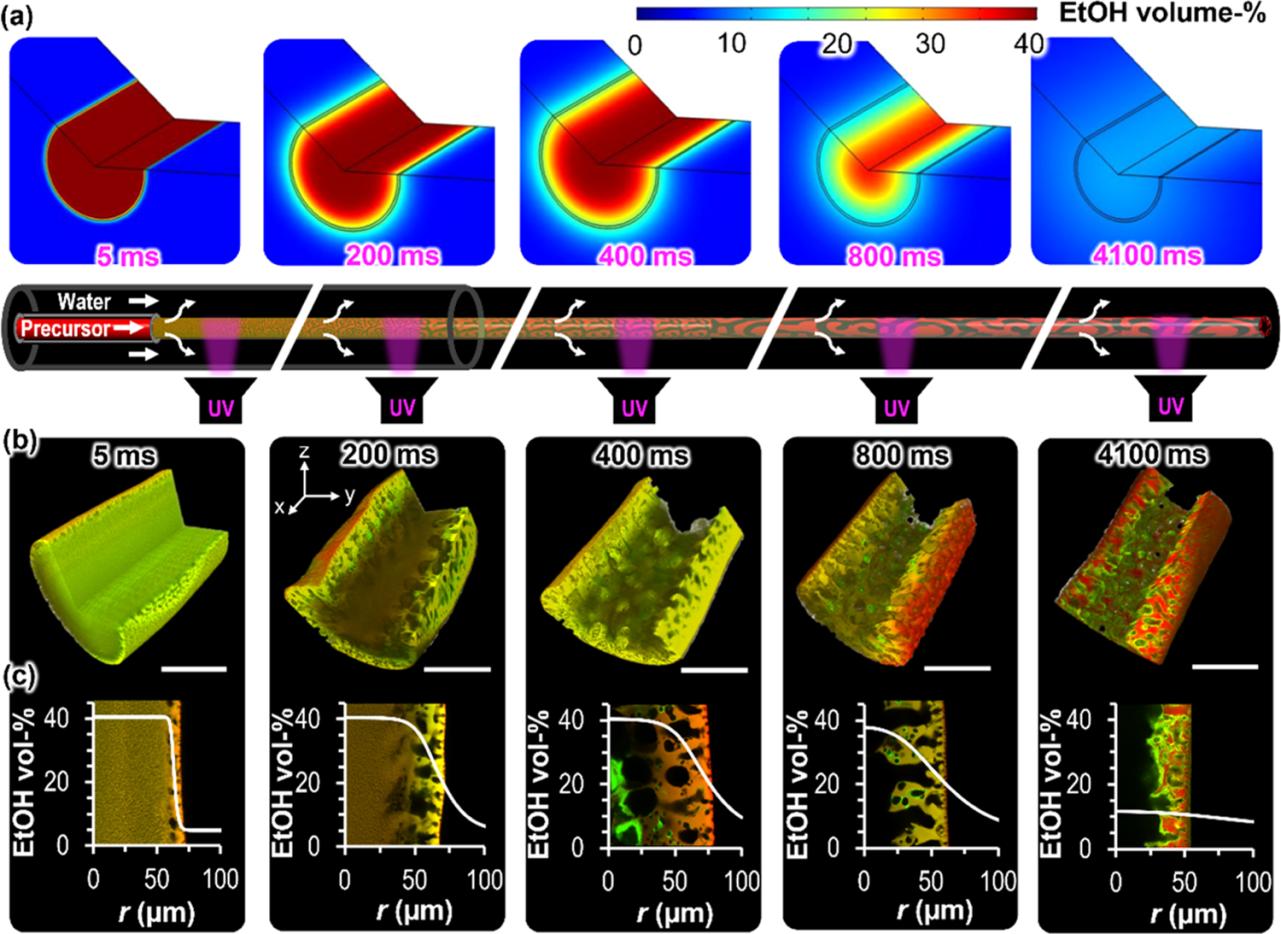
Dynamics of STrIPS and fiber structure evolution. (a) COMSOL surface plots of ethanol concentration (in vol %) in and around the fiber during STrIPS. (b) Confocal microscopy 3D reconstructions of arrested fiber structures of different ages (in milliseconds). (c) Calculated radial ethanol concentration profiles (in vol %) are shown with confocal micrographs of the equatorial fiber plane in the background. All scale bars are 50 μm.

The simulations show that initially the ethanol concentration declines sharply near the surface of the fiber (5 ms, Figure 3a). It takes several hundred milliseconds for the diffusion front to grow inward. After a few seconds, the ethanol concentration within the fiber equilibrates with the surrounding water (≈5 vol %). In the following paragraphs, we compare the fiber structure evolution with the diffusion simulation.

The fiber structure evolution is analyzed by arresting intermediate stages of the phase separation. To this end, the fiber is flown into a rotating water reservoir on a turntable via the microfluidic device (Figure 1b). A beam of UV-light is focused on the fiber at different traveling distances, polymerizing the BDA instantaneously to arrest the corresponding fiber structure. The polymerized fiber samples are treated as described in Section 2.1 and analyzed with the confocal microscope. From the measured fiber extrusion velocity (*u*_fiber_) and UV polymerization distance (*L*_poly_), the age of the fiber is calculated *(t*_fiber_ = *L*_poly_/*u*_fiber_, see SI Section S6 for details). For fibers aged less than 200 milliseconds, the UV polymerization was done after a few millimeters travel distance in the microfluidic channel. Figure 3b shows confocal microscopy 3D reconstructions of fibers of different ages corresponding to different stages of STrIPS.

The phase separation grows from the fiber surface toward the inside. Fibers aged 5 ms show phase-separated structures at the surface but an unstructured interior. After 200 ms, water cavities extend over 50 μm from the fiber surface to the inside, while the fiber center remains unstructured. At 400 ms, the fiber center has also formed large water cavities, which coalesce to form a continuous hollow interior after 800 ms.

The measured fiber structure can be correlated with the local ethanol concentration. To show this, we juxtapose confocal microscopy cross-sections of the fibers with the calculated radial ethanol concentration profiles in Figure 3c. At 5 ms, the ethanol concentration dropped only near the fiber surface, in agreement with the thin phase-separated region near the surface. As the ethanol concentration also starts to decrease below the surface, radial pores extend from the surface inward (200 ms). It takes about 800 ms until also the ethanol concentration in the center decreases, roughly correlating with the onset of phase separation in the fiber middle. When the ethanol concentration in the center reaches ≈10 vol % at 4000 ms, a continuous hollow channel stretches along the length of the fiber.

**Figure 4:**
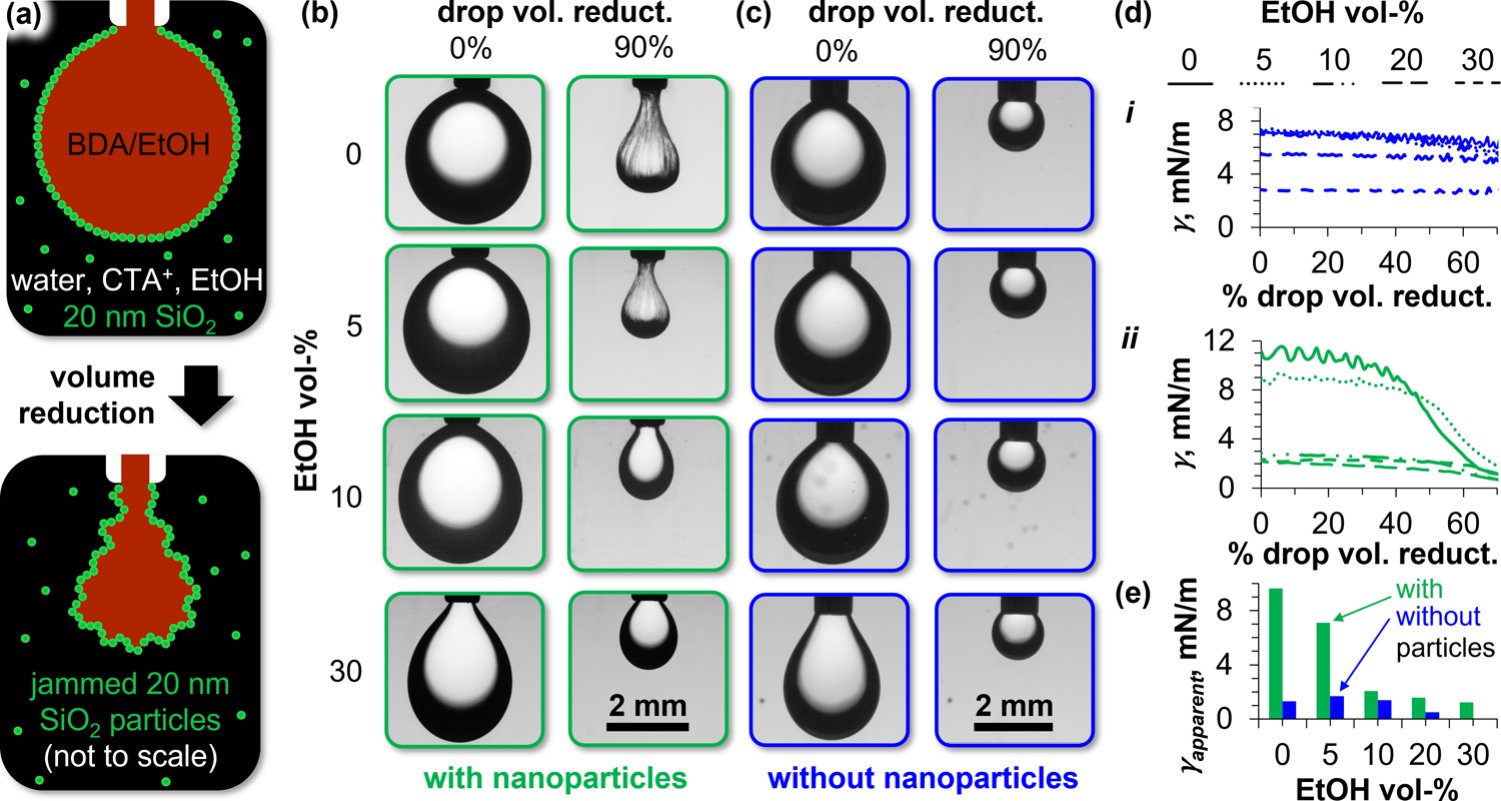
Interfacial elasticity in dependence of ethanol concentration. (a) Schematic depiction of the pendant drop experiment (not to scale). (b) Photographs of pendant BDA droplets at different volume reduction stages (horizontal axis) and variable ethanol volume fractions in the water phase (vertical axis) containing 5 wt % Ludox TMA and 0.5 mM CTA^+^. (c) Photographs of pendant BDA droplets at different ethanol volume fractions in the water phase containing 0.5 mM CTA^+^ and no nanoparticles. (d) Measured interfacial tension γ during shrinking the pendant BDA droplets by 70 vol %, *i* in the absence of particles, and *ii* in the presence of particles. (e) Apparent interfacial tension reduction γ_apparent_ with (green columns) and without (blue columns) nanoparticles.

The hollow interior results from the inability of the Ludox TMA particles to stabilize the BDA/water interface in the center of the fiber. We show that this inability originates from the effect of ethanol on the elastic properties of the interfacial particle film. To this end, a pendant drop of BDA is formed in a colloidally stable aqueous dispersion of Ludox TMA, containing 0.5 mM CTA^+^ and variable amounts of ethanol (Figure 4a). The droplet volume is reduced by 90% via a syringe pump and observed with a camera. Depending on the interfacial behavior of the particles, the droplet interface can wrinkle due to interfacial jamming of the particles (Figure 4a).

Figure 4b shows photographs of pendant BDA droplets covered by Ludox TMA nanoparticles at different ethanol volume percentages before and after 90% volume reduction (see also SI Video 1). As depicted in Figure 4a, for 0 and 5 vol % ethanol, stable wrinkles are observed after 90% droplet volume reduction. These wrinkles show that the nanoparticles can generate a rigid film on the BDA/water interface. In contrast, for ethanol contents above 10 vol %, no wrinkles are observed. The absence of wrinkles indicates that the interfacial film of CTA^+^-modified nanoparticles does not show strong elastic properties above 10 vol % ethanol.

Fitting the droplet with the Young–Laplace equation allows for further analysis of the interfacial elasticity. This analysis assumes that the shape of the droplet results from a balance between the gravitational and interfacial forces. Interfacial tension imposes a spherical droplet shape, while gravity elongates the droplet in vertical direction. With the densities of both water and BDA at their respective ethanol contents (see SI Section S7), the Young–Laplace fit of the droplet yields the interfacial tension γ. When the droplet volume is reduced, the strength of the gravitational force decreases. Figure 4c shows that in the absence of interfacially active particles, the volume reduction results in a more spherical droplet. Figure 4d–*i* confirms that for the droplets in Figure 4c, the Young–Laplace drop shape analysis yields a nearly constant value for γ down to droplet volume reduction of 70% (for volume reductions >70%, the Young–Laplace fit becomes inaccurate due to low Eötvös numbers^33^). In contrast, when reducing the drop volume in the presence of interfacially active particles, the droplet becomes less spherical (Figure 4b). This phenomenon occurs due to the elastic deformation of the interface by the jammed particles. The Young–Laplace analysis interprets the droplet shape transformation in Figure 4b incorrectly as a reduction of γ. This apparent reduction of γ provides a semiquantitative measure of the elasticity of the interfacial particle film,^34,35^ as discussed next.

Figure 4d-*ii* shows the measured γ in dependence of the droplet volume reduction in the presence of nanoparticles for different ethanol concentrations. At 0% ethanol, the initial value of γ is ∼11 mN/m. When the droplet volume is reduced by 70%, the value of γ drops to ∼1 mN/m. We define this difference of γ as the apparent interfacial tension change γ_apparent_ because it does not really represent a reduction of γ, but results from the elastic drop deformation. For different ethanol volume fractions the first thing to note is that the initial values for γ decrease from 11 to 2 mN/m when the ethanol volume fraction is increased from 0 to 30%. The second observation is that for the ethanol volume fractions 0 and 5 vol %, γ drops strongly in response to the shrinking of the droplets, indicating elastic properties of the interfacial particle films. In the presence of nanoparticles, γ_apparent_ decreases with increasing ethanol volume fractions as shown in Figure 4e. This decrease of γ_apparent_ can be interpreted as a weaker elastic response to the droplet shrinking with increasing ethanol volume fraction. In contrast, in the absence of nanoparticles, no significant γ_apparent_ can be observed (the small values of γ_apparent_ ≈ 1 mN/m likely result from inaccurate Young–Laplace fits due to the low Eötvös numbers^33^). We also performed a complementary experiment, which shows that Ludox TMA/CTA^+^-stabilized BDA-in-water emulsions contain nonspherical droplets at low ethanol contents. In contrast, at high ethanol contents, primarily spherical droplets are observed (see SI Section S8). Both, the pendant drop and the emulsification experiments reveal that ethanol reduces the rigidity of the interfacial Ludox TMA/CTA^+^ film.

The reduced nanoparticle film elasticity likely results from the low interfacial tension at elevated ethanol volume fractions. The driving force for the particles to adhere to the interface is their attachment energy Δ*E*_attach_ = −π·*r*^2^·γ·(1–cos θ)^2^ with *r* the particle radius and θ the three phase contact angle.^36^ This simplified formula neglects potential effects arising from line tension.^37^ For θ = 90°, Δ*E*_attach_ = −840 *kT* at 0 vol % ethanol. In contrast, Δ*E*_attach_ = −152 *kT* at 20 vol % ethanol. The higher Δ*E*_attach_ at 0 vol % ethanol suggests that the removal of particles from the interface is obstructed during the droplet volume reduction. Thus, at 0 vol % ethanol, the particles can withstand strong lateral stresses in the interfacial film, resulting in the observed elastic response.

Based on the pendant drop measurements, we can interpret the correlation between the ethanol concentration and the intermediate fiber structures in Figure 3. Near the fiber surface, the rapid decrease of the ethanol concentration enables the particles to generate a rigid interfacial film at early stages of STrIPS. The rigid Ludox TMA/CTA^+^ film stabilizes micrometer-sized BDA/water structures on the fiber surface. In contrast, the prolonged high ethanol concentration in the center of the fiber inhibits the formation of a rigid interfacial film. Thus, the BDA/water interface can coarsen for a longer time, resulting in the formation of a hollow interior of the fiber.

The confocal micrographs in Figure 3c reveal another important aspect: During STrIPS, the particles partition from the BDA-rich phase toward the water. This is indicated by the orange/yellow color of the polyBDA scaffold for times below 800 ms. The color likely originates from the overlap of the Nile red in polyBDA and the green Rhodamine 110 fluorescence of the particles, suggesting that the particles are embedded within the polyBDA. However, after 400 ms, a green fluorescence signal of the particles in the water channel appears, indicating phase transfer of the particles. After 4000 ms, the entire polyBDA scaffold is surrounded by the green fluorescence of aggregated particles in water. This phase transfer of the particles can be controlled by the CTA^+^ functionalization of the particles which depends on the initial CTA^+^ concentration in the fiber casting mixture.

**Figure 5:**
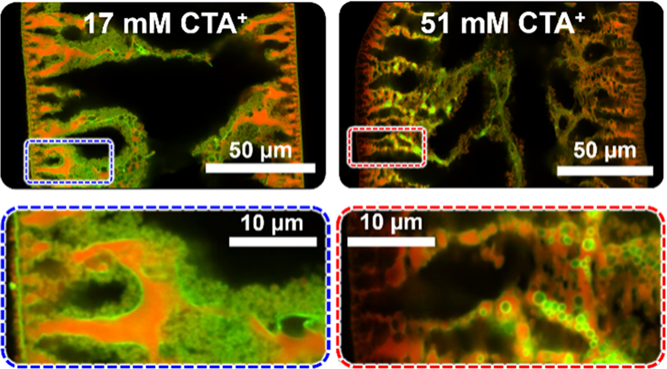
Effect of CTA^+^ concentration on particle partitioning. The confocal micrographs show the equatorial plane and magnified insets of fibers composed of polyBDA (red) and nanoparticles (green).

During STrIPS, the initial CTA^+^ concentration controls whether the particles partition either into the water or whether they remain in the BDA. Figure 5 shows confocal micrographs of cross sections of the final fiber structures (UV-polymerization after 4000 ms) formed with two different CTA^+^ concentrations. The fibers are generated with the liquid composition *iii* near the critical point (Figure 2a). At 17 mM CTA^+^, red fluorescent polyBDA structures are surrounded by a green fluorescent nanoparticle gel in water. In contrast, at 51 mM CTA^+^, the green fluorescence signal originates from the surfaces of water droplets within the polyBDA domains. The formation of these droplets likely takes place via nucleation of water within the BDA-rich phase during STrIPS, as was previously described for ternary liquid mixtures undergoing sequential phase separation events.^38^ Their stabilization by green fluorescent particles indicates that the particles were present in the BDA-rich phase for 51 mM CTA^+^ at the time when the fiber structure was arrested. In contrast, the absence of particle-stabilized droplets in BDA at 17 mM CTA^+^ indicates that the particles have been expelled from the BDA at an earlier stage, as was observed in Figure 3. In the following, we further investigate how the particle partitioning depends on the CTA^+^ concentration for the dynamically evolving composition during STrIPS.

**Figure 6:**
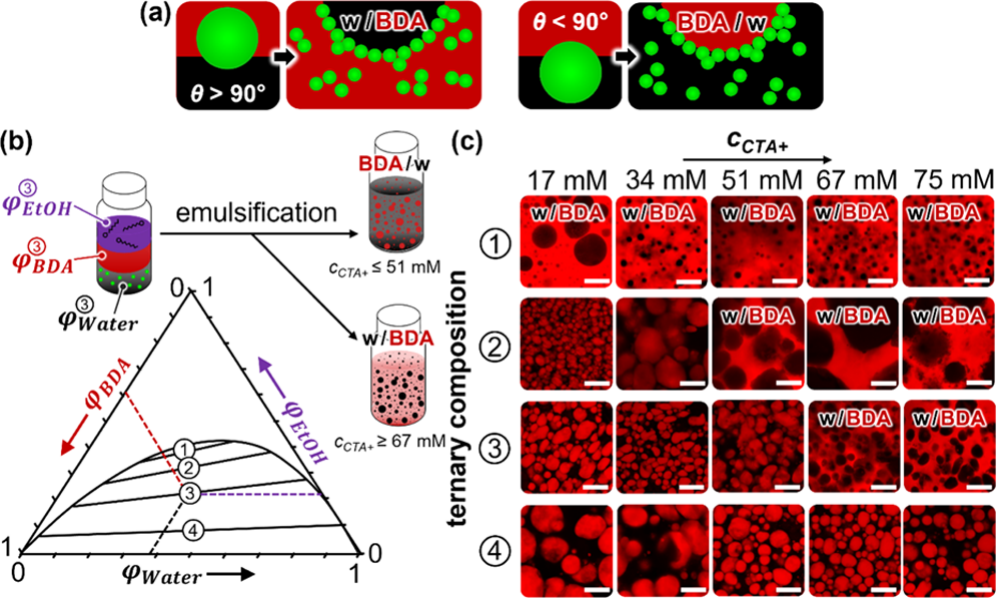
Emulsion inversion during STrIPS. (a) Schematic depiction of particle contact angle θ at a BDA/water interface and effect on the type of emulsion for hydrophobic particles (θ > 90°) and hydrophilic particles (θ < 90°). (b) Ternary phase diagram for BDA/ethanol/water. Particle-stabilized emulsions of BDA/ethanol/water are prepared for midpoint tie-line compositions 1, 2, 3, and 4 (1: φ_BDA_ = 0.32, φ_EtOH_ = 0.38, φ_Water_ = 0.30; 2: φ_BDA_ = 0.34, φ_EtOH_ = 0.32, φ_Water_ = 0.34; 3: φ_BDA_ = 0.38, φ_EtOH_ = 0.23, φ_Water_ = 0.39; 4: φ_BDA_ = 0.45, φ_EtOH_ = 0.07, φ_Water_ = 0.48) using different CTA^+^ concentrations. (c) Confocal microscope images of the emulsions formed with the ternary liquid compositions 1–4 with 17–75 mM CTA^+^. The BDA-rich phase is fluorescently labeled by Nile red. All scale bars are 50 μm.

The extent of CTA^+^ functionalization controls the three phase contact angle θ of the particles (Figure 6a). Hydrophobic particles with θ > 90° partition into the oil (BDA)-rich phase, while hydrophilic particles with θ < 90° partition into the water-rich phase. The former can be distinguished from the latter by testing whether a water-in-BDA (w/BDA) or a BDA-in-water (BDA/w) emulsion is formed for equal volumes of BDA and water.^39^ Here, we employ this experiment to provide additional information on the particle partitioning behavior during the STrIPS process. To this end, we approximate the nonequilibrium compositional evolution during STrIPS with equilibrium mixtures as will be briefly discussed in the following paragraph.

The initial compositions of the BDA- and water-rich phases are given by the compositions *i*, *ii*, or *iii* in Figure 2a. When the fiber enters the stream of water, the diffusion of ethanol shifts the composition below the binodal curve. The ethanol diffusion initializes phase separation, resulting in water- and BDA-rich phases that continue to lose ethanol. The final composition after STrIPS is given by the end points of the tie-line at 5 vol % ethanol because ethanol is infinitely diluted in the large water reservoir containing 5 vol % ethanol (Figure 1b). We estimate the compositional evolution of the BDA- and water-rich phases during this process as two trajectories along the opposite sides of the binodal curve in the ternary phase diagram of Figure 6b. Thus, in the following emulsification experiment, we consider equilibrium compositions of the BDA- and water-rich phases given by the intersects of the binodal curve with the tie-lines. It shall be noted that these equilibrium compositions may not be fully representative for the nonequilibrium process during STrIPS. Nevertheless, our recent work showed that equilibrium compositions can model the nonequilibrium interfacial tension evolution during STrIPS.^22^ Therefore, we prepare equilibrium mixtures with compositions in the middle of the tie-lines (ensuring equal volumes of BDA- and water-rich phases) given by points 1, 2, 3, and 4 in Figure 6b. The mixtures contain the same Ludox TMA and CTA^+^ concentrations as the compositions used to make the fibers. Vigorous agitation of these mixtures results in either w/BDA or BDA/w emulsions. As discussed in the previous paragraph, the resulting emulsion type indicates whether the particles have contact angles θ smaller or larger than 90°.

The preference for the nanoparticles to stabilize either w/BDA or BDA/w emulsions depends both on the tie-line position and the CTA^+^ concentration (*c*_CTA+_). To distinguish between w/BDA and BDA/w emulsions, the BDA phase is fluorescently labeled with Nile red. Confocal microscopy shows black droplets in a red continuous phase for w/BDA emulsions and red droplets in a black continuous phase for BDA/w emulsions. This fluorescent staining method does not allow for direct visualization of the Ludox TMA particles. Figure 6c shows that for all *c*_CTA+_, w/BDA emulsions are observed for tie-line 1. In contrast, for *c*_CTA+_ = 17 and 34 mM, tie-line 2 yields BDA/w emulsions, while for *c*_CTA+_ ≥ 51 mM w/BDA emulsions are observed. At tie-line 3, BDA/w emulsions are formed for *c*_CTA+_ = 17, 34, and 51 mM and w/BDA emulsions are found for *c*_CTA+_ ≥ 67 mM. On the last tie-line 4, all *c*_CTA+_ result in BDA/w emulsions. This dependency indicates that for tie-line 1, the particles are hydrophobic (θ *>* 90°), and that for tie-line 4, the particles are hydrophilic (θ < 90°). At the intermediate tie-lines, *c*_CTA+_ controls whether the particles are hydrophilic or hydrophobic. Accordingly, at tie-lines 2 and 3, higher *c*_CTA+_ renders the particles more hydrophobic.

Based on our approximation that these emulsions made with equilibrium compositions represent the dynamics during STrIPS, the emulsification experiments potentially explain the particle partitioning behavior observed in Figures 3 and 5. Initially, the particles are hydrophobic and partition into the freshly phase-separated BDA-rich phase (Figure 3c, 5–200 ms and Figure 6 tie-line 1). When STrIPS takes place with *c*_CTA+_ ≥ 51 mM, the more hydrophobic character of the particles causes them to reside in the BDA-rich phase to stabilize nucleated water droplets (Figures 5 and 6 tie-line 2). However, at the end of STrIPS, the particles always become hydrophilic and they partition into the water-rich phase (Figure 3c, 4100 ms).

**Figure 7:**
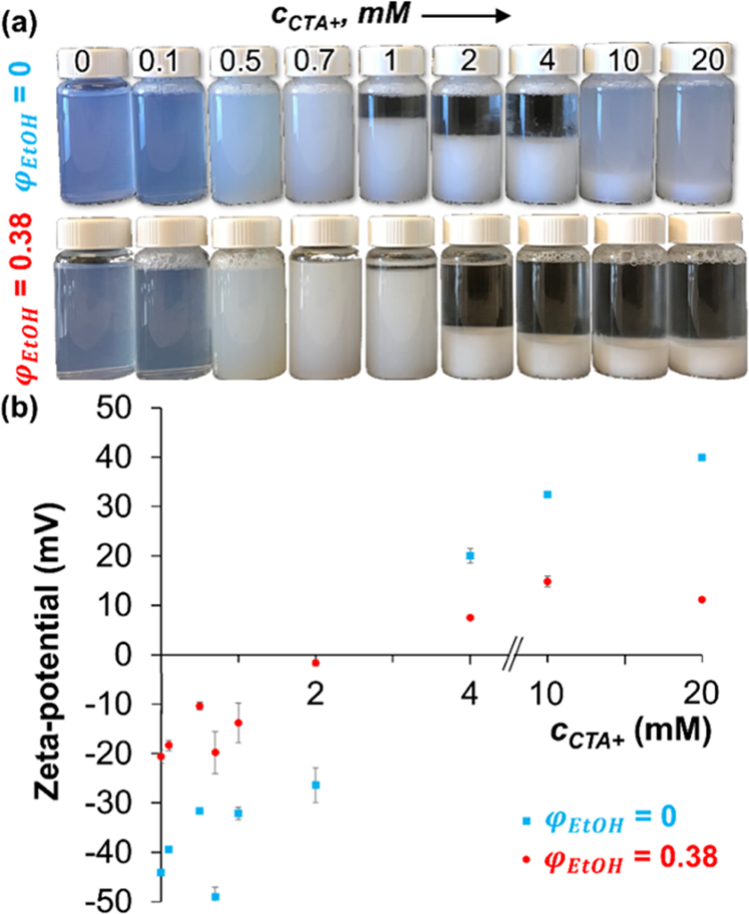
Nanoparticle surface charge. (a) Aqueous dispersions of Ludox TMA particles (pH 3) at variable CTA^+^ concentrations for different ethanol volume fractions φ_EtOH_. (b) ζ-Potential of the particle dispersions as a function of CTA^+^ concentration (measured at 25 °C).

The transition from hydrophobic to hydrophilic particles can potentially be explained by the formation of adsorbed CTA^+^ double layers on the particles. Figure 7 shows photographs and ζ-potential measurements of aqueous Ludox TMA dispersions at different *c*_CTA+_ and two different ethanol volume fractions in the water (φ_EtOH_ = 0 and 0.38). For *c*_CTA+_ < 0.1 mM, colloidally stable dispersions are obtained. However, for *c*_CTA+_ > 0.5 mM, the particles begin to aggregate, visible by the stronger light scattering and sediment formation (*c*_CTA+_ > 1.0 mM). Interestingly, the sediment begins to redisperse at *c*_CTA+_ > 10.0 mM for φ_EtOH_ = 0 (cloudy dispersion above the sediment), while it remains fully aggregated at φ_EtOH_ = 0.38. Complementary ζ-potential measurements show that the particle surface charge becomes less negative as *c*_CTA+_ is increased up to *c*_CTA+_ = 2 mM. For both φ_EtOH_, the ζ-potential becomes positive for *c*_CTA+_ > 4 mM. The charge inversion suggests the formation of CTA^+^ double layers on the particles, as was previously reported.^21,40,41^ The higher positive ζ-potential in the absence of ethanol (φ_EtOH_ = 0) explains the redispersion of the particles for *c*_CTA+_ > 10.0 mM. It can also potentially explain the stronger hydrophilic character of the particles observed for the STrIPS fiber structures (Figure 3c, 4100 ms) and the formation of BDA/w emulsions at tie-line 4 (Figure 6c).^42^

Before concluding the first part of this paper, we briefly mention two preliminary results: (i) As an alternative to ethanol, methanol can be used as a solvent to mix BDA with water. The tie-lines for BDA/methanol/water are more inclined, indicating that methanol preferentially partitions to water compared to BDA (for details, see SI Section S9). The shape of the binodal curve allows us to prepare fiber precursor dispersions with larger BDA volume fractions, resulting in slightly more voluminous polyBDA scaffolds within the fibers. (ii) A variation of the pH value of the aqueous fraction of the fiber precursor dispersion affects the surface charge of the Ludox TMA particles. We find that for both Ludox TMA, as well as Ludox TM particles, this offers some control of the nanoparticle aggregation during STrIPS, but it provides limited control over the final fiber structure (see SI Section S10).

To summarize this section, STrIPS with CTA^+^-functionalized Ludox TMA particles dispersed in ternary mixtures of BDA/ethanol/water or BDA/methanol/water generates fibers with micrometer-sized surface pores and internal pores with sizes of tens of micrometer. The small surface pores result from the fast stabilization of the BDA/water phase separation by nanoparticles as the solvent is rapidly removed near the fiber surface. The large internal pores originate from the delayed solvent removal, impeding interfacial particle stabilization. The delayed diffusion of ethanol from the center of the fiber renders the particles inefficient in arresting the phase separation of BDA and water, resulting in a hollow interior. This hollow interior forms more easily with ethanol in the fiber precursor dispersion, compared to methanol. To obtain hollow fibers, the composition of the fiber precursor dispersion must be near the binodal line of the phase diagram, with a slight offset from the critical point (here φ_BDA_ = 0.367, φ_EtOH_ = 0.418, see Figure 2c-*ii*). Moreover, hollow fibers are successfully formed with aqueous Ludox TMA dispersions at pH 3. Last, the CTA^+^ and nanoparticle concentrations need to be carefully selected to enable the generation of a hollow interior via phase separation (here 17 mM CTA^+^ and 7.8 wt % nanoparticles).

A partial goal of the present study has been to generate hollow fiber membranes with a densely packed coating of nanoparticles on the outer cylindrical surface. Prior research on STrIPS has shown that the addition of nanoparticle contents >25 wt % to the fiber precursor dispersion produces dense coatings on the fiber surface and that this imparts ultrafiltration characteristics to the membrane.^16^ These STrIPS hollow fiber membranes required the injection of an aqueous bore channel into the center of the fiber. Here, we find that adding nanoparticle concentrations of 26 wt % inhibits the formation of a hollow interior via phase separation within the fiber. Instead, the large amounts of particles stabilize a BDA/water scaffold in the center of the fiber (see Figure S13). However, the hollow interior is required as a drainage channel for the ultrafiltration permeate flow. Thus, here we cannot directly synthesize STrIPS ultrafiltration hollow fiber membranes via phase separation and nanoparticle self-assembly alone. Nevertheless, in the next section, we show how the hollow fibers formed here can be postprocessed into ultrafiltration membranes with tunable separation performance.

### 2.3 Membrane Separations with STrIPS Hollow Fibers

**Figure 8:**
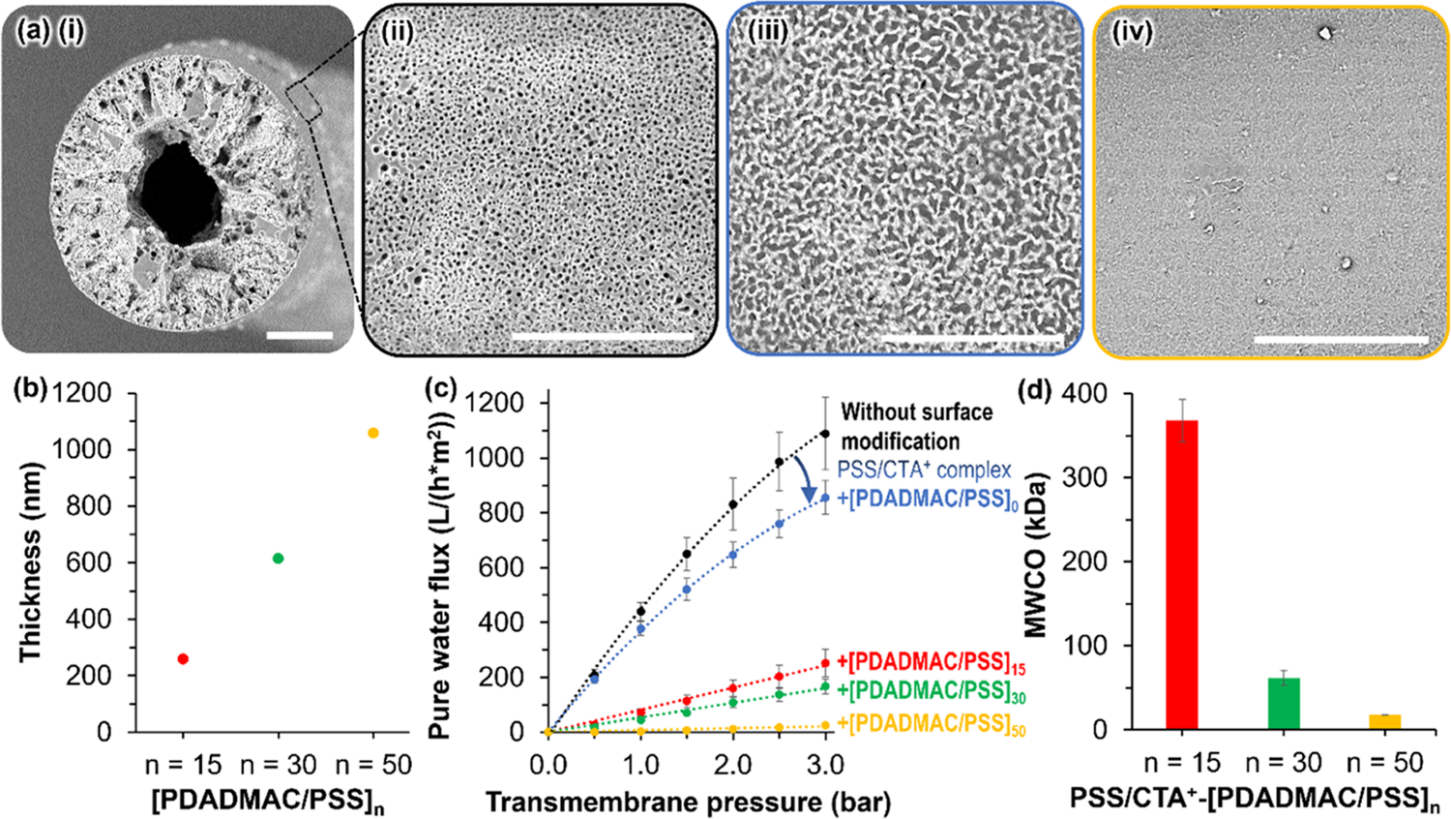
Polyelectrolyte functionalization and aqueous ultrafiltration with STrIPS hollow fiber membranes. (a) Scanning electron microscopy images of (i) the fiber cross section with hollow drainage channel and particle/polyBDA shell, (ii) unmodified fiber surface, (iii) fiber surface after PSS/CTA^+^ complex modification, and (iv) fiber surface after polyelectrolyte coating with 50 bilayers. All scale bars are 50 μm. (b) Dry thickness of PDADMAC/PSS films composed of different numbers *n* of bilayers deposited on silicon wafers. (c) Pure water permeability of the hollow STrIPS fiber membranes before and after PSS/CTA^+^ complexation and for different numbers of PDADMAC/PSS-bilayers. (d) Molecular weight cutoff (MWCO) of the STrIPS hollow fibers treated with PSS/CTA^+^ for different numbers *n* of PDADMAC/PSS-bilayers.

The macroporous morphology of the STrIPS hollow fibers with a mean surface pore size of 1.1 μm suggests their use for microfiltration (Figure 8a-(i) and (ii); for details, see SI Section S12). However, more selective separations can be realized by functionalizing the membrane surface with polyelectrolyte multilayers (PEMs).^25−30^ For the STrIPS fibers, PEM deposition is feasible because the silica nanoparticle decorated membrane surface consists of anionic silanol groups, providing binding sites for the polyelectrolytes. We investigate PEM formation on STrIPS hollow fibers by alternate adsorption of polycations and polyanions. STrIPS membrane separation performance is characterized by pure water permeability (PWP) and molecular weight cutoff (MWCO).

PEMs can control the separation selectivity of membranes by closing pores of the support structure as well as via forming layer-by-layer films on the membrane surface.^43^ PEMs have bilayer thicknesses ranging from only a few nanometers up to tens of nanometers.^44^ Thus, the PEMs are too thin to seal the micron-sized surface pores of the STrIPS membrane here. We therefore plug the surface pores first with a more bulky polyelectrolyte–surfactant complex. To this end, we allow poly(sodium 4-styrene sulfonate) (PSS) and CTA^+^ to form a gel structure within the surface pores of the fiber.

PSS and CTA^+^ can form a water-insoluble complex near and above the critical micelle concentration (cmc) of CTA^+^. To demonstrate this, we mix different aqueous solutions, one containing CTA^+^ at variable concentrations (0.02–10 mM), and the other with PSS kept at a constant molar ratio of 0.001 to the CTA^+^ concentration (2 × 10^–5^–0.01 mM). Below CTA^+^ concentrations of 0.5 mM, these mixtures result in transparent solutions (see SI Figure S18). However, above 0.5 mM, the mixtures display increasing turbidity with increasing CTA^+^ concentration, indicating the formation of the complex. Interestingly, the cmc of CTA^+^ has been experimentally determined to lie between 0.5 and 1.0 mM,^45^ suggesting that the complexation takes place between CTA^+^ micelles and PSS molecules. To seal the micrometer-sized pores of the hollow fibers, the fibers are first soaked in a 5 mM CTA^+^ solution and then submerged in a 1 g/L PSS solution. The SEM micrograph in Figure 8a-(iii) shows that the PSS/CTA^+^ complex changes the fiber surface morphology, demonstrating the coverage of the surface pores.

The surface morphology changes further after PEM functionalization of the fiber as shown in the SEM image of Figure 8a-(iv). We deposit bilayers of poly(diallyldimethylammonium chloride) (PDADMAC) and PSS on top of the PSS/CTA^+^ complex.^43,46,47^ PDADMAC and PSS multilayers are selected for this approach due to their high chemical stability, fitting well with the chemical robustness of the cross-linked polyBDA scaffold of the STrIPS membrane.^48,49^ For the polyelectrolyte coating, the hollow fibers are assembled into a homemade membrane module (for details, see SI Section S15) and alternately immersed in solutions of PDADMAC and PSS (pH 5 and with 500 mM NaCl) until films of 15, 30, and 50 PDADMAC/PSS-bilayers are formed. Ellipsometry measurements shown in Figure 8b indicate that the polyelectrolytes produce PEMs with a dry thickness of up to 1000 nm for 50 PDADMAC/PSS-bilayers (see the Experimental Section for details). The swelling of PDADMAC/PSS in water further increases the multilayer thickness by more than 40%,^50,51^ indicating that the PEMs close the STrIPS fiber surface pores.

The pure water permeability (PWP) of the hollow fibers decreases after PSS/CTA^+^ complexation and PEM deposition (Figure 8c). Unmodified STrIPS hollow fibers have a PWP of approximately 390 ± 40 L/(h*m^2^*bar) which drops to 310 ± 20 L/(h*m^2^*bar) after PSS/CTA^+^ complexation. This permeability loss suggests that the PSS/CTA^+^ complex plugs the fiber surface pores (see Figure S19). Figure 8c furthermore shows that the PWP curve flattens with increasing transmembrane pressure. This nonlinear PWP dependence likely results from a compaction of the porous membrane structure for elevated pressures, narrowing the smallest fiber pores. The PWP is further reduced to 7 ± 1 L/(h*m^2^*bar) after deposition of 50 bilayers of PDADMAC/PSS (PSS/CTA^+^-[PDADMAC/PSS]_50_) on the fiber surface. For the PEM-coated hollow fibers, the water flux correlates linearly with the transmembrane pressure. This suggests that the PEM introduces a significant hydraulic resistance to the membrane which controls the membrane selectivity, as will be discussed in the next paragraph.

The PEM deposition is accompanied by a change in the molecular weight cutoff (MWCO) of the STrIPS fibers (Figure 8d). The MWCO is defined as the approximate molecular weight of the solute which is rejected by the membrane to a level of 90%.^52^ For the STrIPS fibers covered with a PSS/CTA^+^ complex alone, no MWCO can be detected since dextran molecules of 6–650 kDa still permeate through the membrane. After 15 bilayers of PDADMAC/PSS, however, ultrafiltration properties are introduced to the STrIPS membrane with an MWCO of 368 ± 26 kDa. The membrane pore size is thus controlled by the number PDADMAC/PSS multilayers. For PSS/CTA^+^-[PDADMAC/PSS]_30_ the MWCO decreases to 62 ± 9 kDa, and to 18 ± 0.2 kDa for PSS/CTA^+^-[PDADMAC/PSS]_50_. These MWCOs are still substantially higher than those observed for membranes where the PEM coating becomes the active separation layer, clearly indicating a pore narrowing effect where a thicker PEM leads to a smaller pore size.

The MWCO can be used to estimate the mean pore size of the STrIPS hollow fiber membrane after PEM modification. This relation can be described by *d*_P_ = 0.11 × MW^0.46^ with *d*_P_ the hydraulic pore diameter (nm) and MW the molecular weight of the Dextran according to the MWCO (Da).^53^ Following the MWCO, the maximum pore sizes amount to 40 nm for PSS/CTA^+^-[PDADMAC/PSS]_15_, 18 nm for PSS/CTA^+^-[PDADMAC/PSS]_30_, and 10 nm for PSS/CTA^+^-[PDADMAC/PSS]_50_. Comparing the estimated pore size with the thickness of one PDADMAC/PSS bilayer of around 20 nm for the present experimental conditions suggests that the polyelectrolyte film is not evenly coating the pores of the STrIPS membrane. Viewing the roughness of the porous membrane, smaller surface pores can be covered and closed by the PEM and the PSS/CTA^+^ complex, whereas bigger pores are more likely narrowed to an extent that depends on the initial pore size.

Nevertheless, combining a PSS/CTA^+^ complex with a PDADMAC/PSS multilayer shifts the separation selectivity of the STrIPS hollow fiber membrane from micro- to ultrafiltration. A further decrease of the MWCO can potentially be accomplished by terminating the PDADMAC/PSS multilayer with a thin separation layer of a densely packed polyelectrolyte combination.^54^ To further improve control over the STrIPS membrane selectivity, more research is needed on forming smaller and more uniform STrIPS membrane pores which can be efficiently closed by thinner PEMs.

## 3 Conclusions

In this article, we investigate the mechanisms and dynamics in structure evolution of nanocomposite hollow fiber membranes formed via solvent transfer induced phase separation (STrIPS). Microfluidic fiber spinning of a homogeneous precursor dispersion composed of butanediol diacrylate (BDA), ethanol, and water with CTA^+^-functionalized Ludox TMA particles produces hollow fibers with micrometer-sized surface pores. Confocal microscopy analysis combined with solvent diffusion modeling and elasticity measurements of the interfacial nanoparticle film reveal that the small surface pores result from the fast stabilization of the BDA/water phase separation. The delayed solvent removal from deeper within the fiber impedes interfacial particle stabilization, resulting in a hollow interior. We show that the filtration selectivity of these hollow fiber membranes can be tailored from micro- to ultrafiltration. Therefore, the STrIPS fiber surfaces are functionalized with a complex of a cationic surfactant with an anionic polyelectrolyte, followed by the deposition of polyelectrolyte multilayers. These combined STrIPS membrane modifications efficiently tune the pure water permeability and the pore size of the membrane. Producing polyelectrolyte multilayers of different thicknesses enables the removal of dextran > 360 kDa, > 60 kDa, and > 18 kDa. The findings of this work improve the understanding of STrIPS and demonstrate the utilization of STrIPS hollow fibers for molecular separations. Based on the simplicity of the membrane spinning process, our work can inspire more research on STrIPS nanocomposite membranes for advanced separation applications.

## 4 Experimental Section

### 4.1 Hollow Fiber Precursor Preparation

The hollow fiber precursor dispersion consists of a homogeneous mixture of 1,4-butanediol diacrylate (BDA, 36.7 vol %; ChemCruz), ethanol (41.8 vol %; Merck), demineralized water (21.5 vol %; MilliQ Water Purification), Ludox TMA nanoparticles (7.8 wt %; Grace), and hexadecyltrimethylammonium cations (CTA^+^, 17 mM; Sigma-Aldrich). A detailed composition of all STrIPS fiber casting mixtures is given in the Supporting Information (Figure S2). The photo-initiator 2-hydroxy-2-methylpropiophenone (Sigma-Aldrich) is added to 0.3 vol % to the casting mixtures to polymerize the BDA monomers after phase separation. Visualization of the oil phase is accomplished by fluorescence labeling with Nile red (Sigma-Aldrich). All chemicals purchased are of analytical grade.

Ludox TMA nanoparticles are prepared by concentrating the particle stock dispersion from 34 to 45 wt % by the evaporation of water. The concentrate is centrifuged to remove nanoparticle aggregates (15 min at 2500 rpm; Microfuge 16 Beckman Coulter), adjusted to pH 3 by the addition of 1 M HCl (Acros Organics), and then dialyzed in water containing 50 mM NaCl (Merck).

### 4.2 Fiber Preparation via STrIPS

For microfluidic fiber spinning the precursor dispersion is pumped at a rate of 80–120 μL/min through a 100 μm capillary orifice into a stream of water, flowing in a coaxially aligned outer capillary (1 mm inner diameter; World Precision Instruments) at a rate of 1–1.5 mL/min (Figure S5). All inner glass surfaces of the capillaries are coated with a solution of 0.2 wt % poly(diallyldimethylammonium chloride) (PDADMAC; Sigma-Aldrich) and 500 mM NaCl to prevent adhesion of the precursor. The fibers are collected in a slowly rotating water bath of pH 3 containing 5 vol % ethanol and exposed to a beam of high-intensity UV light (5 min) to obtain solid Ludox TMA/polyBDA composite fibers.

### 4.3 Structure Characterization

The STrIPS fiber structure is analyzed by confocal laser scanning microscopy (Stellaris 5, Leica Microsystems). The polymerized fibers are washed in a solution of 50 vol % 1 M HCl and 50 vol % ethanol to remove CTA^+^. After immersion in an alkaline Rhodamine 110 (Chemodex) solution, the fibers are made optically transparent by replacing water with diethyl phthalate (Acros Organics). Upon excitement with 488 nm laser light, the Rhodamine 110 labeled particles emit green fluorescence detected at 500–550 nm, while the Nile red fluorescence of the polyBDA is detected at 590–700 nm (Figure S7). The fiber surface is imaged via scanning electron microscopy (SEM; Phenom ProX, Thermo Fisher Scientific) applying an electron beam excitation of 10 kV and a 3 nm layer of sputter-coated platinum.

For studying the emulsification behavior during STrIPS, immiscible BDA/ethanol/water liquid mixtures are prepared with a constant amount of 7.8 wt % particles. The dispersions containing Nile red in the BDA phase are mixed and filled into a 1 mm glass capillary (Vitrocom) to avoid solvent evaporation during confocal analysis. To minimize the effect of the glass surface on the phase separation behavior, the capillaries are coated with 0.2 wt % PDADMAC and 500 mM NaCl.

### 4.4 Transient Solvent Diffusion Modeling

A transient solvent diffusion simulation is employed to obtain the concentration profiles for ethanol for the extrusion of the hollow fiber precursor dispersion into water. A COMSOL Multiphysics model (version 5.5) with the Physics engine “Transport of Diluted Species” is designed for the present flow geometry, dimensions, and velocities. The model couples flow and ethanol diffusion from the precursor dispersion to the surrounding water and neglects convection of water as radial diffusion dominates over axial dispersion (see SI of ref (23)). We further refine the transient diffusion model by considering concentration-dependent diffusion coefficients and a thin diffusion barrier on the surface of the fiber to account for the smaller fiber surface pores (for details, see SI Section S6).

### 4.5 Pendant Drop Experiments

20 mL solutions of CTA^+^ (5 mM), water, Ludox TMA (5 wt %), and ethanol (0, 5, 10, 20, 30, 40 vol %) are mixed. The dispersions are tip-sonicated for 30 s (30% amplitude of a 500 W tip sonicator, 10 mm tip diameter; Qsonica LCC) to redisperse aggregated silica particles. Separately, 10 mL solutions of water and ethanol (0, 5, 10, 20, 30, 40 vol %) are prepared to which 2 mL of BDA is added. These solutions are shaken vigorously. Subsequently, the BDA and water are separated via centrifugation at 1000 rpm for 15 min (Microfuge 16 Beckman Coulter) and decantation. The densities of the BDA phases as well as the Ludox dispersions are determined with a calibrated pipette and an analytical balance (AG204 DeltaRange). A droplet of the BDA phase (15 μL) that has been equilibrated with the alcoholic solutions of the respective ethanol vol % is formed in the aqueous dispersion of Ludox TMA and CTA^+^ with the corresponding ethanol volume fraction. Videos of the droplet shape during volume reduction (90 vol %) are recorded on a pendant drop tensiometer (Dataphysics OCA25). The drop shapes are analyzed with the ImageJ plugin pendent drop.^55^

### 4.6 ζ-Potential Measurements

Dispersions (20 mL) are prepared by mixing Ludox TMA particles (5 wt %; brought to pH 3 by the addition of 1 M HCl), CTA^+^ (0–20 mM), water, and ethanol (0 and 38 vol %). The solutions are sonicated for 20 min in an ultrasonication bath (Branson 1800). After resting for 1 h at room temperature, the samples are vortexed for 30 s (Scientific Industries VORTEX Genie 2) and the ζ-potential of the dispersions is measured as triplicate at 25 °C (Zetasizer Ultra, ZSU5700, Malvern Pananalytical).

### 4.7 Polyelectrolyte Multilayer Coating

For PEM coating, hollow STrIPS fibers of 200 μm diameter are fabricated by extrusion into water as described in Section 4.2 (using a 200 μm tapered, round cross-section capillary). The fibers are wetted for 60 min in a 5 mM CTA^+^ solution (pH 9) after extrusion and polymerization. The fibers are then submerged for 60 min in a 1 g/L solution of poly(sodium 4-styrene sulfonate) (PSS, 200 000 g/mol; Sigma-Aldrich) of pH 5 and 500 mM NaCl. The high coating salinity is chosen to obtain a larger increase in thickness per coating step. The fibers are assembled in a homemade membrane module (Figure S20), equilibrated to the ionic strength of 500 mM NaCl at pH 5 and then immersed in a 1 g/L solution of PDADMAC (200 000–350 000 g/mol; Sigma-Aldrich) of pH 5 and 500 mM NaCl for 2 min. The fibers are rinsed for 2 min with 500 mM NaCl solution of pH 5. To complete one cycle of bilayer, fibers are immersed for 2 min in a solution of 1 g/L PSS adjusted to pH 5 and 500 mM NaCl, followed by 2 min of rinsing with 500 mM NaCl of pH 5. This procedure is repeated until the desired number of bilayers is reached. The coated fibers are rinsed with 100 mL of demineralized water for 2 h. Sets of fibers assembled in three different modules are coated according to this procedure.

The PEM thickness is derived from ellipsometry measurements. To this end, silicon wafers with a native silicon oxide layer of 2.3 nm are coated with 15, 30, and 50 bilayers of PDADMAC/PSS, respectively. The measurements are performed on a rotating compensator ellipsometer (Mk-2000 V, J.A. Woollam Co.) followed by data analysis using the CompleteEASE software package (J.A. Wollam Co.). Triplicate samples are measured across a wavelength range of 370–1000 nm at incidence angles of 65, 70, and 75° under ambient conditions.

The dry thickness of the PEM is obtained by data fitting to a standard Cauchy model according to eq 1. Here, *n* is the refractive index of the polyelectrolyte layer, λ is the incidence wavelength, and *A*, *B*, and *C* represent the Cauchy parameters.
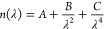
1

### 4.8 Hollow Fiber Performance Testing

The hollow fiber separation performance is evaluated before and after polyelectrolyte modification by measuring pure water permeability (PWP) and molecular weight cutoff (MWCO) in a homemade membrane module. For a permeability test, the membrane module is connected to a pressurized water reservoir. After equilibrating water flow through the module for 1 h at a transmembrane pressure of 0.5 bar, water flux is measured over a pressure range of 0.5–3 bar by the water permeated through the fibers for 3 min. The PWP follows from the slope of a plot of water flux versus transmembrane pressure. Figure S20 shows the experimental setup for the hollow fiber performance testing.

The MWCO for the hollow fiber membranes is determined using a series of aqueous 1 g/L dextran solutions of molecular weight in the range of 6–650 kDa (6, 15–25, 40, 70, 100, 450–650 kDa; Sigma-Aldrich). The feed solution is flown through the fibers at a transmembrane pressure of 2 bar, and after each dextran filtration run, the fiber module is rinsed with water. Dextran concentrations in the feed (*C_f_*) and in the permeate (*C*_p_) are determined by gel permeation chromatography with a size exclusion column (Agilent 1200/1260 Infinity GPC/SEC series, Polymer Standards Service data center and column compartment). Solutions are flown over two Polymer Standards Servie Suprema 8 × 300 mm^2^ columns in series (1000 Å, 10 μm followed by 30 Å, 10 μm) at 1 mL/min, and dextran concentrations are determined via refractive index measurements. The solute rejection for each dextran fraction is given by *R*_D_ = (1 – *C*_p_/*C*_f_) × 100%. The dextran rejection is plotted as sieving curve to determine the MWCO of the STrIPS membrane (Figure S21). Both, PWP and MWCO are determined from fiber sets assembled in three different membrane modules.

## Supporting Information Available

Pendant drop shrinkage with nanoparticles (SI Video 1) (MP4)
Pendant drop shrinkage without nanoparticles (SI Video 2) (MP4)
Measurement of ternary phase diagrams; fiber precursor dispersion preparation; microfluidic device assembly; microfluidic fiber spinning; fluorescence emission spectra of Rhodamine 110 chloride and Nile red; transient solvent diffusion modeling; pendant drop experiments; droplet shape analysis; methanol as solvent for STrIPS; STrIPS fibers with Ludox nanoparticle dispersions at different pH; fiber internal surface area analysis; surface pore size distribution of STrIPS hollow fiber membrane; PSS/CTA^+^ complexation; polyelectrolyte functionalization of STrIPS hollow fibers; and STrIPS hollow fiber testing module; membrane separations with STrIPS hollow fibers (PDF)
