# Supplementary material for: Synthesis and Polyelectrolyte Functionalization of Hollow Fiber Membranes Formed by Solvent Transfer Induced Phase Separation
Source: ACS Appl Mater Interfaces. 2022 Sep 15;14(38):43195–206. doi: 10.1021/acsami.2c10343 (PMC9523618; doi:10.1021/acsami.2c10343)
Supplement: Supplementary file 3 — am2c10343_si_003.pdf [file am2c10343_si_003.pdf]

Supporting Information

Synthesis and polyelectrolyte functionalization of hollow fiber membranes formed by solvent transfer induced phase separation

Henrik Siegel[1]*, Alessio J. Sprockel[1], Matthew S. Schwenger[2], Jesse M. Steenhoff[1], Iske Achterhuis[3], Wiebe M. de Vos[3], Martin F. Haase[1]*

[1] Van't Hoff Laboratory of Physical and Colloid Chemistry, Department of Chemistry, Debye Institute for Nanomaterials Science, Utrecht University, 3584 CH Utrecht, The Netherlands

[2] Rowan University, Henry M. Rowan College of Engineering, Glassboro, NJ, 08028, USA

[3] Faculty of Science and Technology, Membrane Surface Science, Membrane Science and Technology, MESA+ Institute of Nanotechnology, University of Twente, 7500 AE Enschede, The Netherlands

* Correspondence: h.siegel@uu.nl; m.f.haase@uu.nl

Table of contents

S1. Measurement of ternary phase diagrams S3
S2. Fiber precursor dispersion preparation S4
S3. Microfluidic device assembly S5
S4. Microfluidic fiber spinning S7
S5. Fluorescence emission spectra of Rhodamine 110 chloride and Nile red S8
S6. Transient solvent diffusion modelling S8
S7. Pendant drop experiments S10
S8. Droplet shape analysis S10
S9. Methanol as a solvent for STrIPS S12
S10. STrIPS fibers with Ludox® nanoparticle dispersions at different pH S12
S11. Fiber internal surface area analysis S15
S12. Surface pore size distribution of STrIPS hollow fiber membrane S17
S13. PSS/CTA$^+$ complexation S18
S14. Polyelectrolyte functionalization of STrIPS hollow fibers S19
S15. STrIPS hollow fiber testing module S20
S16. Membrane separations with STrIPS hollow fibers S20
Supplementary References S21

S1. Measurement of ternary phase diagrams

The binodal curves of the ternary phase diagrams for BDA/ethanol/water and BDA/methanol/water are determined by turbidimetry. Water is added to a miscible mixture of BDA and solvent (ethanol or methanol) until the mixture becomes cloudy. For this transition from mixed clear compositions to turbid demixed compositions the weight fractions of all phases are determined on a mass balance. The weight fractions are converted into volume fractions using the density of BDA (ρ = 1.01 g/mL), water (ρ = 0.998 g/mL), ethanol (EtOH; ρ = 0.77 g/mL), and methanol (MeOH; ρ = 0.77 g/mL; Fig. S1). The experimental procedure is illustrated in detail in the SI of reference [1] (p. 5-7). Table S1 reports the liquid volume fractions φ for the mixing/demixing transitions for BDA/ethanol/water and BDA/methanol/water to determine the binodal curves, respectively.

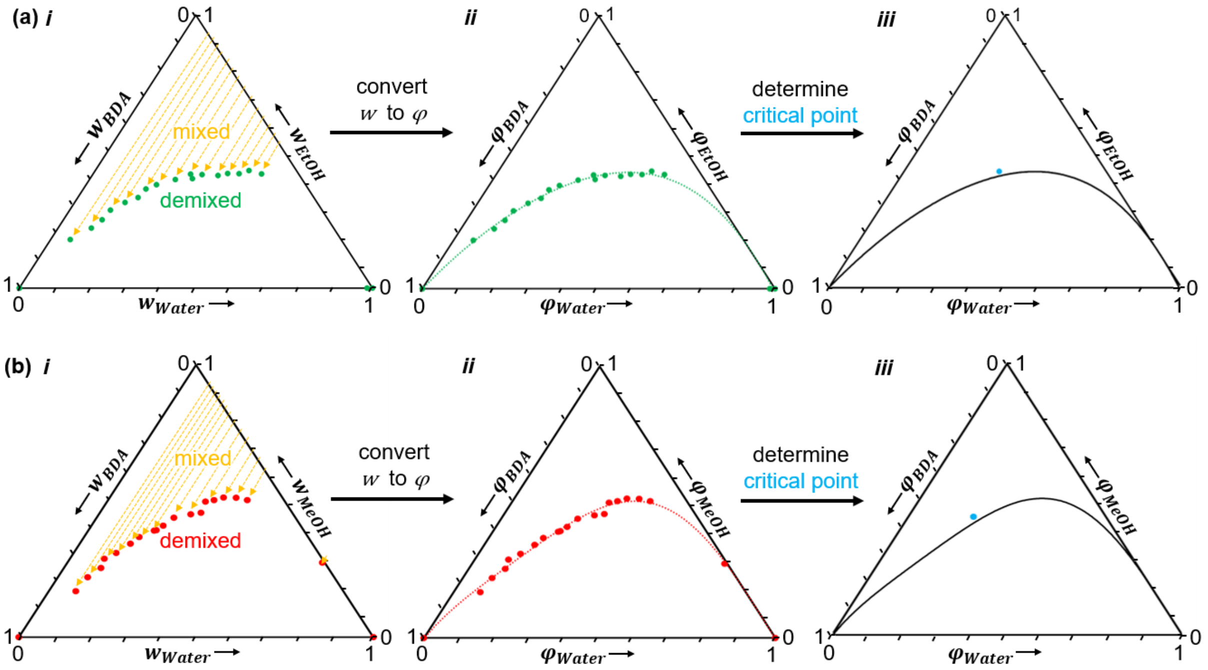

Figure S1: Measurement of the ternary phase diagrams of (a) BDA/ethanol/water and (b) BDA/methanol/water. *(i)* Green and red dots represent the liquid weight fractions *w* for which demixing of BDA, water and solvent is observed. *(ii)* Conversion of the liquid weight fractions into volume fractions φ and fitting of the binodoal line. *(iii)* Determination of the critical point (blue) by checking the emulsion inversion along the binodal line.

The critical point of the ternary phase diagram is assigned to the ternary liquid composition on the binodal where emulsion inversion occurs from water-in-BDA to BDA-in-water. Following this, the critical point is determined for BDA/ethanol/water as φ_{BDA} = 0.301, φ_{EtOH} = 0.427, φ_{Water} = 0.272, and for BDA/methanol/water as φ_{BDA} = 0.377, φ_{MeOH} = 0.436, φ_{Water} = 0.187. Knowledge of the critical point also allows for graphical tie-line construction illustrating the BDA/water demixing process (see SI of reference [1]; p. 5-7). The resulting ternary phase diagrams for BDA/ethanol/water and BDA/methanol/water are shown in Fig. S2.

Table S1: Liquid volume fractions φ for the demixing of homogeneous ternary liquid mixtures of BDA/ethanol/water and BDA/methanol/water.

φ(BDA)	φ(EtOH)	φ(Water)	φ(BDA)	φ(MeOH)	φ(Water)
0.9994	0.0000	0.0006	0.7550	0.1687	0.0763
0.7664	0.1780	0.0556	0.6957	0.2202	0.0841
0.6859	0.2197	0.0943	0.6412	0.2543	0.1045
0.6402	0.2506	0.1093	0.6148	0.2884	0.0968
0.5986	0.2859	0.1155	0.5704	0.3095	0.1202
0.5435	0.3139	0.1427	0.5133	0.3431	0.1436
0.4976	0.3315	0.1709	0.4746	0.3679	0.1557
0.4600	0.3626	0.1774	0.4222	0.3913	0.1864
0.4220	0.3789	0.1991	0.3879	0.4093	0.2028
0.3593	0.3979	0.2428	0.3427	0.4391	0.2183
0.3058	0.4175	0.2767	0.2890	0.4515	0.2595
0.3095	0.4010	0.2895	0.2579	0.4567	0.2854
0.2746	0.4154	0.3100	0.2243	0.4979	0.2778
0.2311	0.4125	0.3564	0.1971	0.5038	0.2991
0.2000	0.4195	0.3804	0.1656	0.5131	0.3212
0.1680	0.4190	0.4130	0.1313	0.5120	0.3567
0.1345	0.4308	0.4347	0.1039	0.5034	0.3927
0.1047	0.4185	0.4768	0.0072	0.2735	0.7193
0.0153	0.0000	0.9847	0.4155	0.3930	0.1915

S2. Fiber precursor dispersion preparation

Homogeneous STrIPS fiber precursor dispersions are prepared using 1,4-butanediol diacrylate (BDA), water and solvent (ethanol or methanol) at compositions listed in Fig. S2. Miscibility of the precursor dispersions is dictated by the binodal curve of the ternary phase diagram (Fig. S2). The hollow fiber precursor composition is provided by number 2 in Fig. S2-(a).

The Ludox® TMA particles are added to the precursor mixtures according to the weight fractions in Fig. S2. These particle weight fractions represent 40 wt-% of the water volume fraction φ_{Water} of each fiber precursor dispersion. Hexadecyltrimethylammonium cations (CTA^+) are dissolved in solvent (ethanol or methanol) and added as 200 mM stock solution to the fiber precursor dispersions.

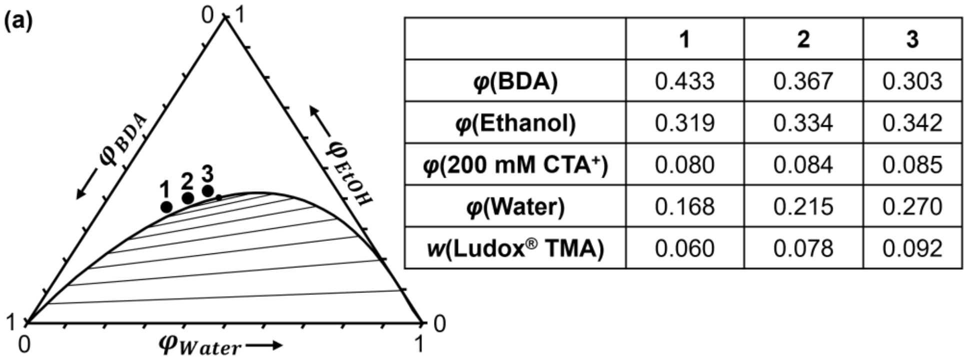

	1	2	3
**φ(BDA)**	0.433	0.367	0.303
**φ(Ethanol)**	0.319	0.334	0.342
**φ(200 mM CTA⁺)**	0.080	0.084	0.085
**φ(Water)**	0.168	0.215	0.270
***w*(Ludox® TMA)**	0.060	0.078	0.092

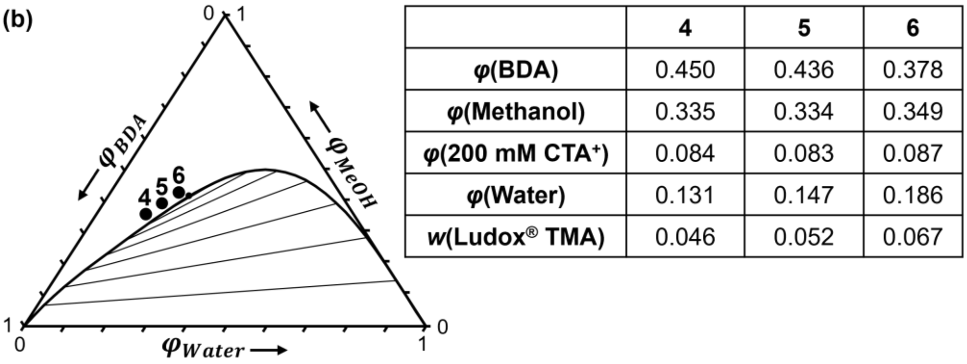

	4	5	6
**φ(BDA)**	0.450	0.436	0.378
**φ(Methanol)**	0.335	0.334	0.349
**φ(200 mM CTA⁺)**	0.084	0.083	0.087
**φ(Water)**	0.131	0.147	0.186
***w*(Ludox® TMA)**	0.046	0.052	0.067

Figure S2: (a) Ternary phase diagram for BDA/ethanol/water with STrIPS fiber precursor compositions numbered as 1, 2 and 3; (b) Ternary phase diagram for BDA/methanol/water with STrIPS fiber precursor compositions numbered as 4, 5 and 6. Liquid volume fractions are expressed by φ, and w represents the particle weight fraction in the precursor dispersion.

S3. Microfluidic device assembly

The microfluidic device is assembled on a microscope slide (25 mm x 70 mm; Epredia) and is composed of a tapered round cross section capillary and a square capillary (Fig. S3).

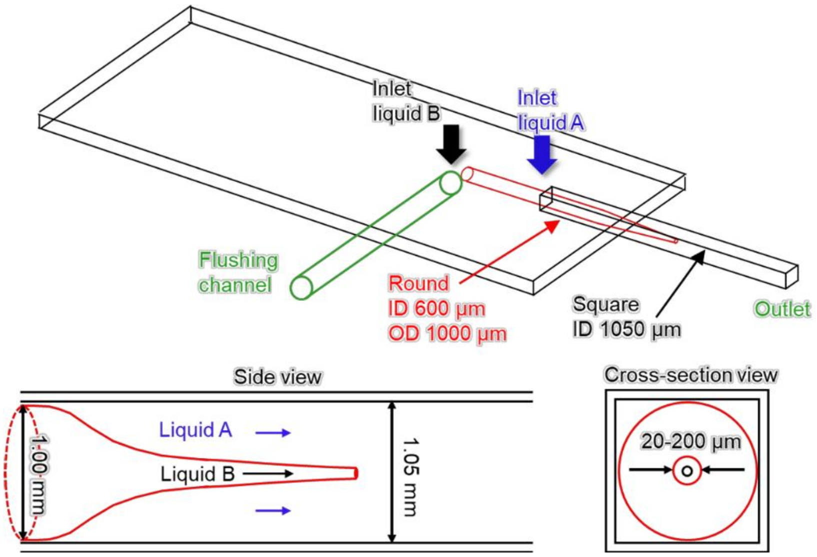

Figure S3: Schematic of microfluidic fiber spinning device.

The first assembly step involves the gluing of a 2-3 cm long segment of the square capillary (custom made capillary from Vitrocom, ID 1.05 mm) onto the microscope slide (Fig. S4(a), (b)). To this end, 5 minute two-component epoxy glue (Liqui Moly) is used. We employ the capillary puller Sutter P1000 to taper a 1 mm outer diameter round capillary (ID 0.6 mm, Fig. S4(c)). The long taper of the round capillary is opened via scoring and breaking the glass with the help of a Sutter scoring

tile. The tapered round capillary is then placed inside the square capillary as shown in Fig. S4(d)-(f). Next, two dispensing needles (OKI TE Needle 20 GA 1/2”; Metcal) are shortened with a razor blade and placed onto the openings of the square and round capillaries (Fig. S4(g)-(i)). Additionally, a metal needle is placed underneath the dispensing needle in the back. The dispensing needles are glued onto the microscope slide with the Epoxy glue. The glue is allowed to cure for 2 hours before the microfluidic device is used for fiber spinning.

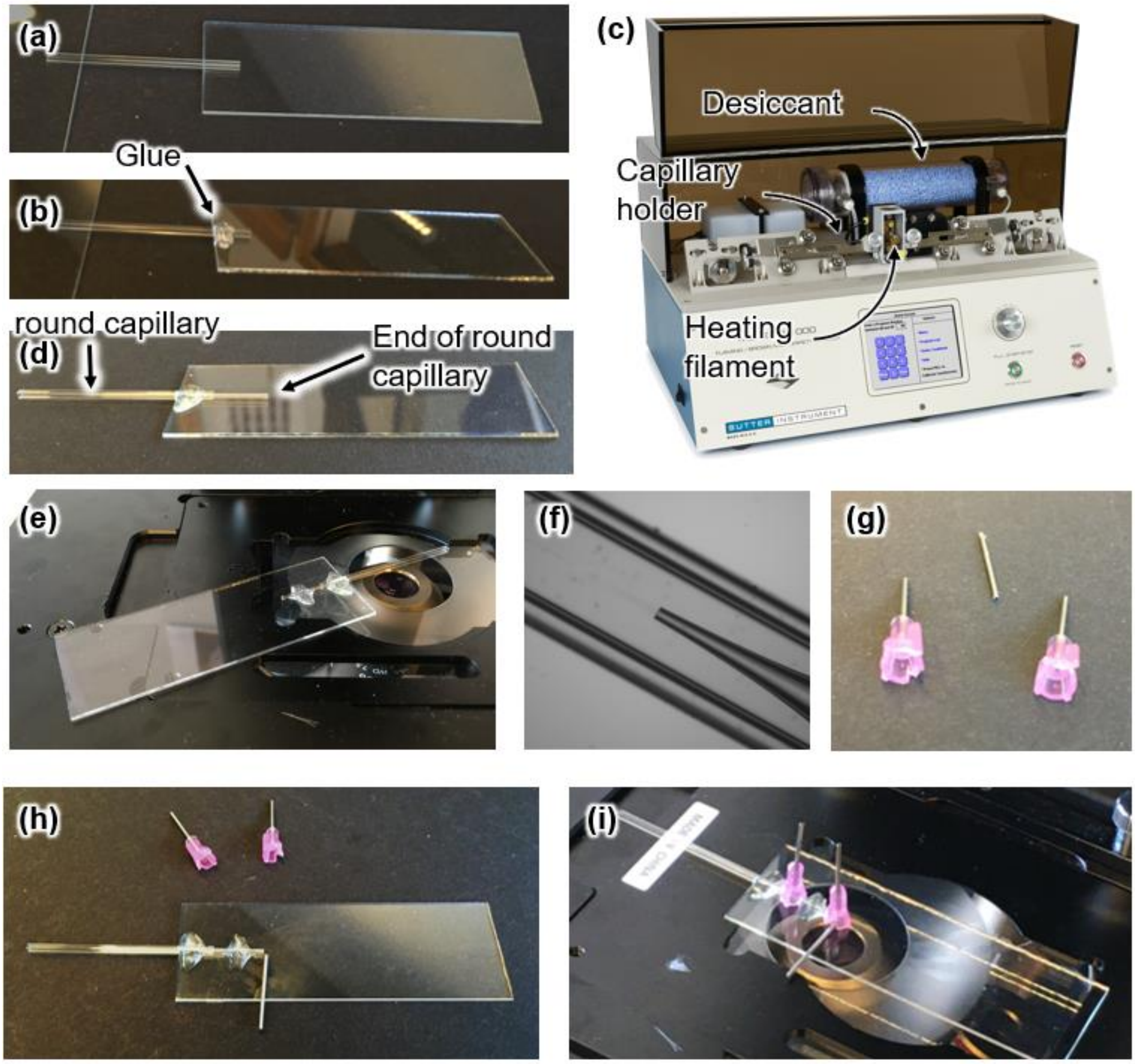

Figure S4: Photographs of the sequential steps of microfluidic device assembly. (a) Square capillary on microscope slide; (b) Glued square capillary; (c) Sutter P1000 capillary puller; (d) Tapered round capillary inserted into square capillary; (e) Gluing of round capillary, device on microscope stage; (f) Micrograph of the end of the tapered round capillary in the square capillary; (g) Dispensing needles; (h) Needle as flushing channel on microscope slide; (i) Assembled device before last Epoxy gluing step.

S4. Microfluidic fiber spinning

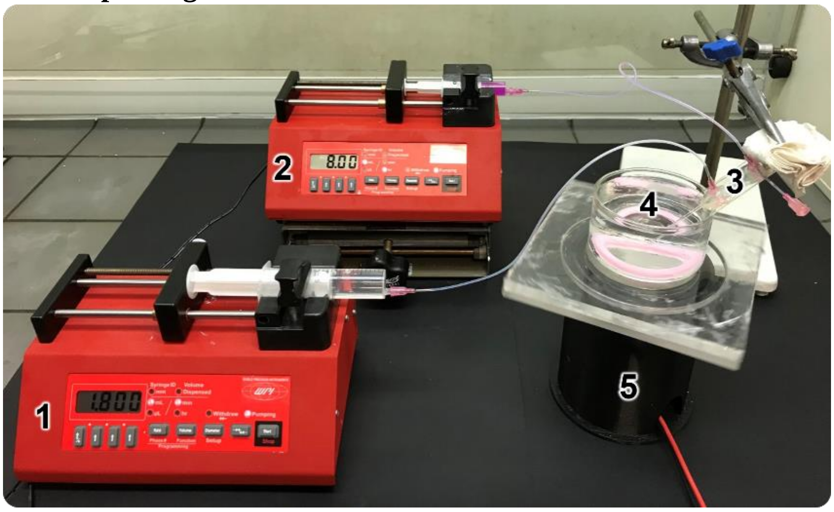

Figure S5: Microfluidic fiber spinning using syringe pumps to flow a continuous water stream (1) and the hollow fiber precursor dispersion (2) through the microfluidic device (3). The fiber is collected in a water bath (4) installed on a turntable support (5). The UV-lamp is not included.

To validate the reproducibility of the microfluidic fiber spinning process Fig. S6 shows micrographs of three fiber replicates manufactured from the same precursor composition as the hollow fiber (Fig. 2(c)-*ii* in the manuscript). All equatorial sections of the fiber samples comprise the same structure features such as the macroporous fiber shell, a hollow interior, and a thin nanoparticle film around the oil domains. This highlights the uniformity of the fiber structures obtained from the spinning process.

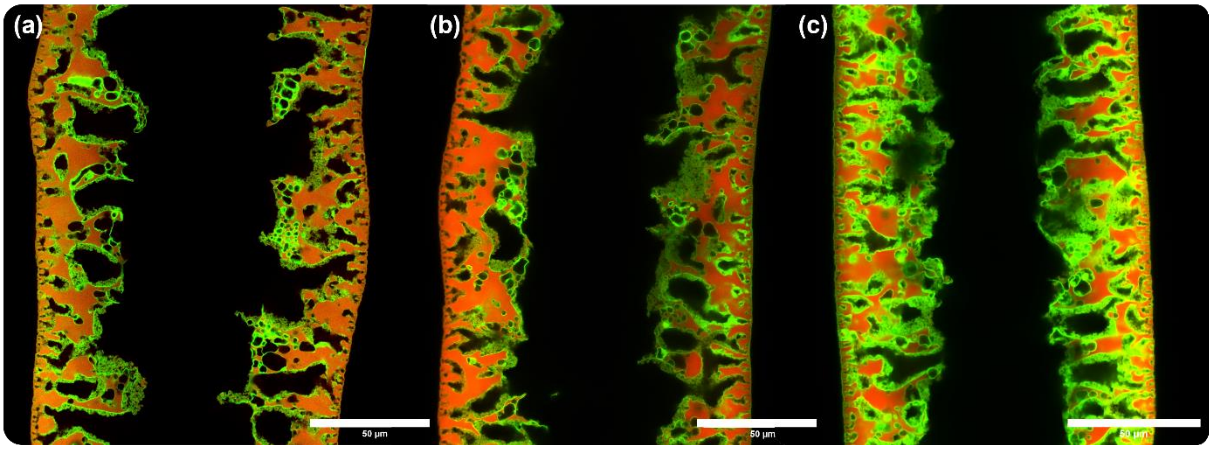

Figure S6: Confocal micrographs from the equatorial plane of STrIPS fiber replicates (a), (b) and (c) manufactured from the precursor composition of the STrIPS fiber in Fig. 2(c)-*ii* in the manuscript. PolyBDA is labelled in red, water in black, and the nanoparticles in green. Scale bars 50 µm.

S5. Fluorescence emission spectra of Rhodamine 110 chloride and Nile red

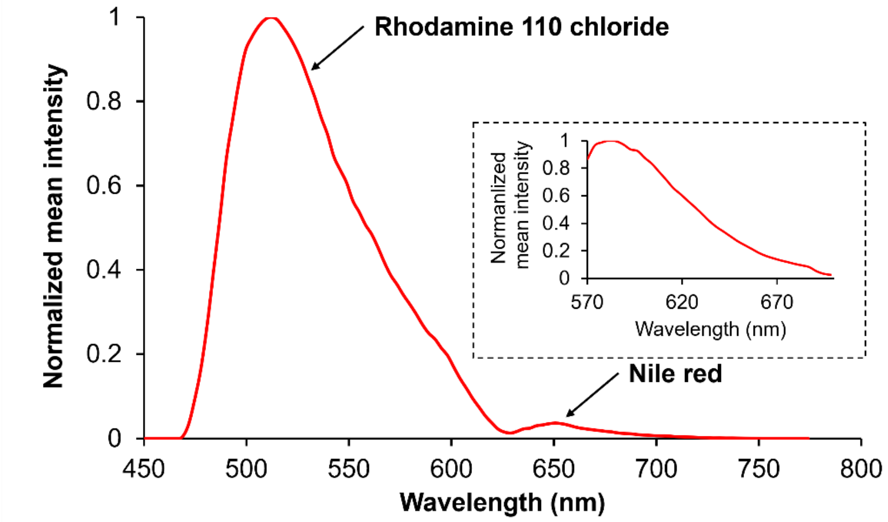

Figure S7: Normalized fluorescence emission spectra of Rhodamine 110 chloride exhibiting a maximum peak intensity at 515 nm and shouldered peak of Nile red with a maximum fluorescence intensity at 650 nm. The fluorescence intensity spectrum is acquired at 488 nm laser light excitation by local probing of a fluorescence dye-labelled fiber immersed in diethyl phthalate. The inset shows the fluorescence emission of Nile red upon excitation with 561 nm laser light.

S6. Transient solvent diffusion modelling

A COMSOL® solvent diffusion model is correlated with the confocal micrographs of STrIPS fiber segments that were UV-polymerized after different travelling distances (Fig. 3 in the manuscript). In the COMSOL® simulation radial diffusion dominates the solvent transport over axial dispersion as shown in detail in the SI of reference [2] (p. 18-19). The fiber travelling time before polymerization (t_{fiber}) is calculated from the fiber extrusion velocity (u_{fiber}) and the fiber travelling distance (L_{poly}, see Table S2) which is determined by the position of the UV-lamp in the extrusion container (Eq. 1). Like this, u_{fiber} follows from the extrusion flow rate of the fiber precursor Q (5 mL/h) divided by the area of the round capillary nozzle A (of radius r = 50 µm):

$$t_{fiber} = \frac{L_{poly}}{u_{fiber}} = \frac{L_{poly} * A}{Q} = \frac{L_{poly} * \pi * r^2}{Q} \quad (1)$$

Table S2: Fiber travelling distance before UV-polymerization (L_{poly}) and corresponding travelling time (t_{fiber}).

**L_{poly} (mm)**	2	80	140	250	895
**t_{fiber} (ms)**	5	200	400	800	4100

The COMSOL® model uses Fick's second law of diffusion, describing the ethanol concentration c_E in the precursor dispersion and the surrounding water with the differential equation Eq. 2:

$$\frac{\partial c_E}{\partial t} = D_E \frac{\partial^2 c_E}{\partial r^2} \quad (2)$$

Axis-symmetric cylindrical coordinates are used to describe the fiber position. To reflect the geometries of microfluidic fiber spinning a cylinder with a diameter of 100 µm is placed inside a larger cylinder of 1000 µm. The inner cylinder represents the precursor dispersion, the outer cylinder is the water filled glass capillary into which the dispersion flows. At the beginning of the simulation (t = 0 ms), the inner cylinder has an ethanol concentration of $c_E^0 = \frac{\varphi_E^0 \cdot \rho_E}{M_E} = \frac{0.418 \cdot 790 \frac{kg}{m^3}}{0.046 \frac{kg}{mol}} = 7169 \frac{mol}{m^3}$. The outer cylinder has an initial ethanol concentration of 835 mol/m^3 (corresponding to 5 vol-%). We refine the transient diffusion model by considering (i) concentration dependent diffusion coefficients and (ii) a thin diffusion barrier on the surface of the fiber to model the smaller surface pores.

(i) The concentration dependence of the diffusion coefficient D_E for ethanol in water is plotted in Fig. S8(b) [3], [4]. For the diffusion simulation, D_E is evaluated via linear interpolation function between the data points in Fig. S8(b) to solve Eq. 3:

$$\frac{\partial c_E}{\partial t} = D_E(c_E) \frac{\partial^2 c_E}{\partial r^2} \tag{3}$$

(ii) The STrIPS fibers typically form a surface with a lowered porosity compared to the fiber interior. This radial pore size gradient is incorporated in the model as thin diffusion barrier on the fiber surface. We therefore assume over a thickness of 3 µm below the fiber surface a diffusion coefficient of $D_E' = D_E(c_E)/3$ [1].

Viewing the only slight incline of the tie-lines in the ternary phase diagram of BDA/ethanol/water (Fig. S2(a)) it is assumed that the partitioning coefficient for ethanol between the BDA- and the water-rich phase is 1.

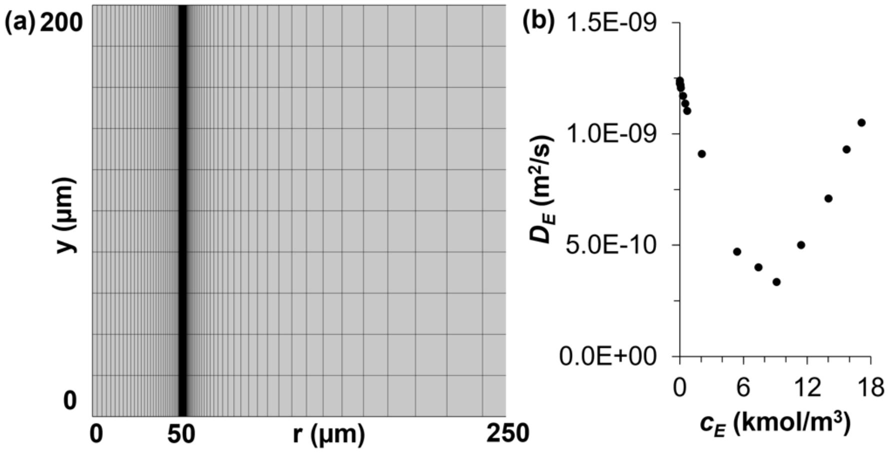

Figure S8: (a) Mesh representing nodes for the numerical calculation of the ethanol concentrations in the simulation with cylindrical positions r and y. (b) Diffusion coefficients D_E for ethanol in water in dependence of the ethanol concentration c_E.

S7. Pendant drop experiments

The samples without the nanoparticles are prepared in the same way and using the same compositions as the samples with Ludox® TMA particles (as described in section 4.5 in the manuscript). Before drop shrinking, the droplets equilibrated to the ambient phase for 20 min. Table S3 lists the densities of the BDA and the water phase containing different volume fractions of ethanol.

Table S3: Densities of water and BDA phases prepared with different ethanol concentrations (reported in volume-%).

Densities of water and BDA for pendant drop experiment **with** nanoparticles							
Ethanol volume-% in water	vol-%	0	5	10	20	30	40
BDA (incl. ethanol)	g/mL	1.057	1.058	1.032	1.041	1.023	1.005
water (incl. ethanol)	g/mL	1.028	1.026	1.020	1.026	0.992	0.970
Densities of water and BDA for pendant drop experiment **without** nanoparticles							
Ethanol volume-% in water	vol-%	0	5	10	20	30	40
BDA (incl. ethanol)	g/mL	1.055	1.057	1.052	1.050	1.034	1.019
water (incl. ethanol)	g/mL	0.998	0.987	0.979	0.976	0.957	0.940

S8. Droplet shape analysis

The shape of the Ludox® TMA/CTA^+ stabilized droplets of BDA in water is analyzed for different ethanol contents in the emulsion. To this end, emulsions with water as continuous phase, 20 vol-% BDA and 0, 20, and 35 vol-% ethanol are prepared. All liquid mixtures contain 5 wt-% Ludox® TMA particles and 0.5 mM CTA^+. The BDA-phase is green labelled with the fluorescence dye Coumarin 6 (Sigma Aldrich). For emulsification the samples are mixed with an Ultra-Turrax® (IKA® T25 digital) at 20000 rpm for 25 seconds. Brightfield images of the particle/surfactant-stabilized emulsions are acquired at 40x magnification (Nikon Eclipse Ts2 with 8 MP CCD Thorlabs microscopy camera) (Fig. S9). The droplet sphericity is derived from confocal analysis. Rectangular capillaries (ID 0.1 mm, length 20 mm; Vitrocom) are filled with the emulsion and confocal images are taken at 100x magnification (470 nm laser excitation line; 300 ms exposure time; 100 % laser intensity; 525 nm detection line; Nikon Ti-2 Eclipse). Confocal images are acquired at five different positions in the capillary. Representative confocal micrographs of the BDA droplets in water are shown in Fig. S9(b).

To analyze the droplet circularity the confocal images are processed with the software Fiji ImageJ (version 1.53k14). First, the color/brightness of the images is adjusted followed by thresholding to highlight the droplets from the dark background. The images are binarized and the pixel roughness around the edges of the droplet is smoothened to avoid any bias in the circularity analysis. Droplet agglomerates are separated using the watershed function. Finally, the scale of the images is set to the pixel-aspect ratio of 11.0934 pixels/µm. The circularity of the droplets is determined using the “Analyze particle” feature. The droplet circularity is classified as 0-0.1, 0.11-0.2, 0.21-0.3, 0.31-0.4, 0.41-0.5,

0.51-0.6, 0.61-0.7, 0.71-0.8, 0.81-0.9, and 0.91-1.0. The circularity is calculated according to equation 4. To exclude interference of pixel artefacts in the analysis a diameter of 1 µm is chosen as minimum droplet size. Furthermore, droplets lying at the edges of the image are excluded from analysis.

$$Circularity = \frac{4\pi * A}{p^2} \tag{4}$$

With A the droplet area (µm^2) and p the droplet perimeter (µm). The circularity distribution of the BDA droplets is shown as histogram in Fig. S9(c). The histogram is obtained from counting the number of droplets per circularity class. Since the number of droplets varies between the confocal images the histogram shows the relative frequency of a circularity class. The relative frequency is the number of droplets that have been assigned to a certain class compared to the total number of droplets per sample. A total of five confocal images is analyzed per emulsion sample. Like this, 178 droplets are characterized for the emulsion formed with 0 vol-% ethanol, 1168 droplets for the emulsion containing 20 vol-% ethanol and 769 droplets for the emulsion containing 35 vol-% ethanol.

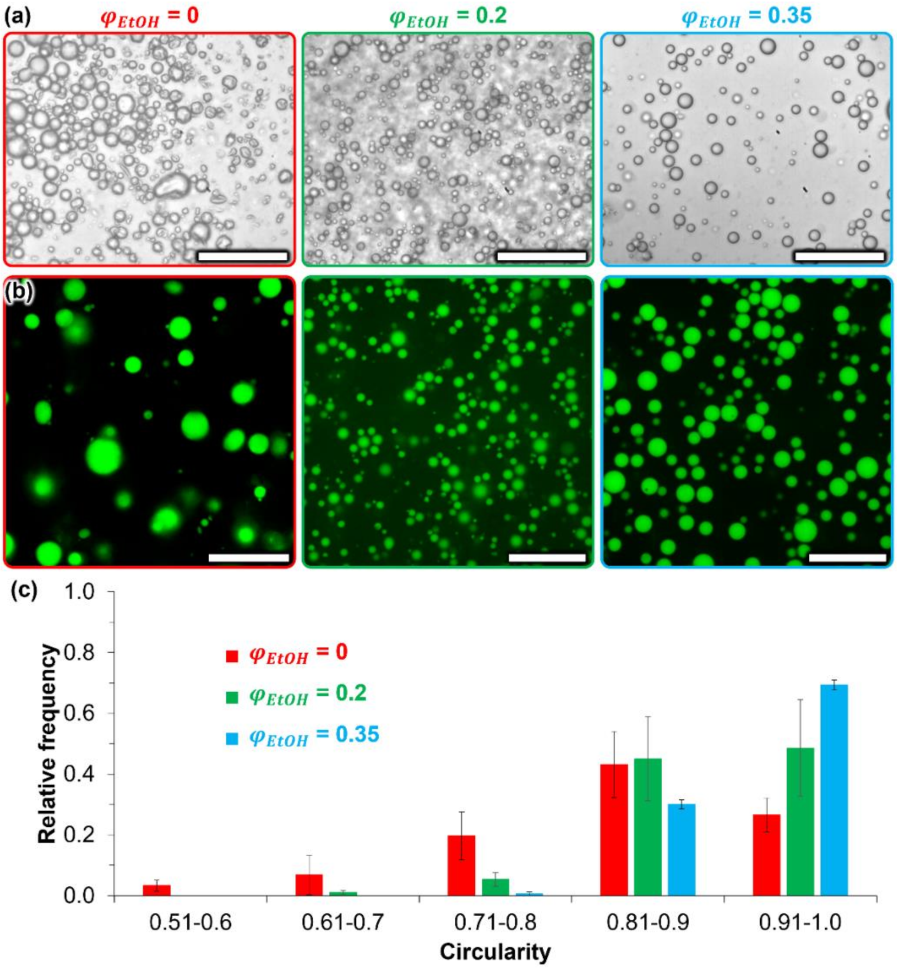

Figure S9: (a) Brightfield microscopy images of Ludox® TMA/CTA$^+$ stabilized droplets of BDA in water (40x magnification). The emulsions are prepared with different ethanol volume fractions φ_{EtOH}. Scale bars 50 µm. (b) Confocal microscopy images of Coumarin 6-labelled BDA droplets in water (100x magnification). The BDA-rich phase is green, the water-rich phase is black. Scale bars 50 µm. (c) Histogram of the relative frequency of the circularity of the BDA droplets in water. Each bar represents the average frequency determined from the analysis of five emulsion samples, with error bars showing the standard deviation.

Fig. S9(c) shows that with increasing ethanol concentration more droplets of a spherical shape (circularity 0.91-1.0) are found. In the absence of ethanol (φ_{EtOH} = 0) non-spherical BDA-in-water droplets occur more prominently. For all ethanol concentrations, no droplets of circularities < 0.5 have been found.

S9. Methanol as a solvent for STrIPS

Alternatively to ethanol, methanol can be used as solvent to fabricate STrIPS nanocomposite fibers with structures shown in Fig. S10.

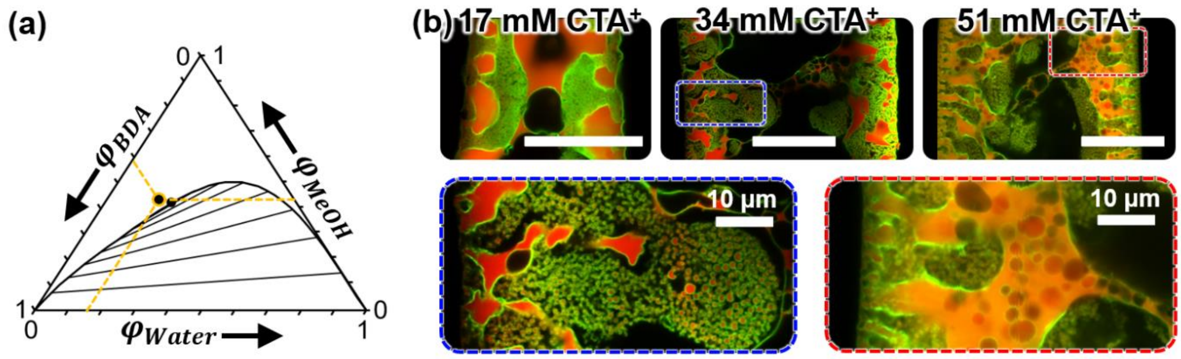

Figure S10: (a) Ternary phase diagram of BDA/methanol/water highlighting the composition used to make STrIPS fibers (b) with different CTA^+ concentrations. The confocal micrographs show the equatorial plane and magnified insets of fibers composed of polyBDA (red), water (black), and nanoparticles (green). Scale bars 50 µm unless specified as 10 µm.

The critical point for the BDA/methanol/water liquid system contains a larger BDA volume fraction of φ_{BDA} = 0.38 as compared to BDA/ethanol/water (φ_{BDA} = 0.30). Thus, fibers made with BDA/methanol/water have more voluminous polyBDA domains. Another difference between the structures formed with BDA/methanol/water is that the aggregated Ludox® TMA particles have distributed more within the water domains of the fibers. In the inset of 34 mM CTA^+ (Fig. S10(b)), it can be observed that the particles are present as interfacial films on polyBDA spheres in the water. These spheres have likely formed via nucleation and growth of BDA droplets, which were subsequently stabilized by the CTA^+ functionalized particles. At 51 mM CTA^+ also particle stabilized water droplets within the polyBDA domains occur. Thus, solvent polarity as well as CTA^+ functionalization affect the particle distribution and aggregation in STrIPS fibers.

S10. STrIPS fibers with Ludox® nanoparticle dispersions at different pH

We studied the STrIPS fiber structure for two types of nanoparticles. STrIPS fibers are prepared using (i) Ludox® TMA nanoparticles with a silica core and Al-incorporations at the particle surface and (ii) pure silica Ludox® TM50 particles. Both nanoparticle species are spherical with a particle radius of 10

nm but have different surface charge densities (Fig. S11): for pH > 4 of the Ludox® TMA dispersion the Al-incorporations lower the negative particle surface charge compared to Ludox® TM50. Fibers are extruded from a homogeneous BDA/ethanol/water mixture (composition 3 of Fig. S2) containing 17 mM CTA^+. Confocal micrographs of the equatorial plane of the fibers are shown in Fig. S11.

STrIPS fibers with Ludox® TMA particles dispersed at pH 7 show a polyBDA shell which is structured by radial water channels and droplet-shaped water enclosures. These nucleated and anisotropic features disappear for fibers fabricated with the same particles dispersed at pH 3 and 6 (magnified insets in Fig. S11). For Ludox® TM50 large particle clusters stretch along the fiber interior with a pronounced anisotropic shell structure at pH 7.

The negative surface charge of the Ludox® particles results from the deprotonation of the silanol groups at the particle surface, and for Ludox® TMA also arises from the dissociation of protons from Al-groups. For increasing pH of the particle dispersion more silanol groups deprotonate, allowing for more CTA^+ molecules to adsorb on the particle surface [5]. The higher surface charge density of TM50 particles compared to Ludox® TMA at neutral pH potentially allows more CTA^+ adsorption on the TM50 species. This difference in particle hydrophobization potentially explains the large Ludox® TM50 aggregates observed in these fibers compared to fibers manufactured with Ludox® TMA particles.

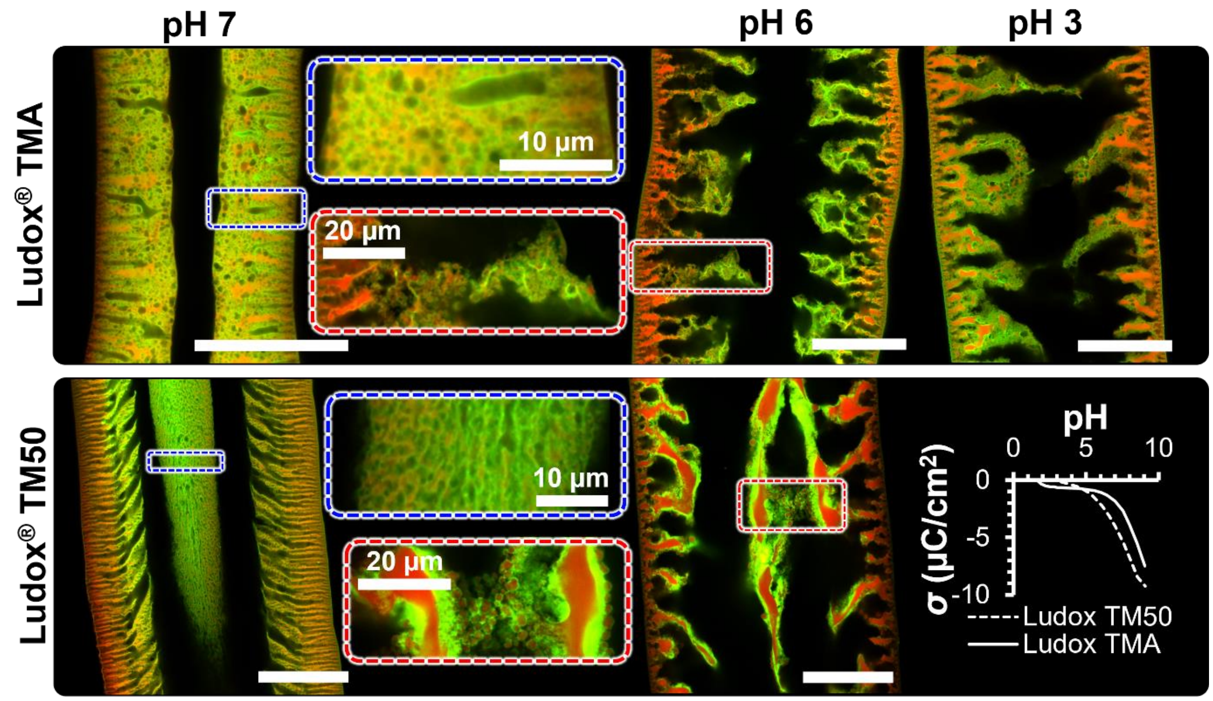

Figure S11: STrIPS fibers fabricated with Ludox® TMA and Ludox® TM50 nanoparticles. The confocal micrographs and the magnified insets show the equatorial plane of fibers composed of polyBDA (red) and nanoparticles (green), extruded from a BDA/ethanol/water liquid mixture (composition 3 of Fig. S2) with 17 mM CTA^+. No colloidal stable precursor mixture of BDA/ethanol/water is obtained with Ludox® TM50 particles dispersed at pH 3. The inserted plot shows the surface charge density σ of Ludox® TMA and Ludox® TM50 particles against pH of the particle dispersion. Scale bars 50 µm unless otherwise specified.

The surface charge density of the Ludox® TMA and Ludox® TM50 particles is derived from titration experiments. 1 M HCl is added to 40 mL Ludox® particle stock dispersion under continuous stirring until pH 2.5 is reached (pH(Ludox TMA® stock dispersion) = 5.23; pH(Ludox® TM50 stock dispersion) = 9.08). After each addition endpoint measurements are performed when the pH has stabilized (Mettler Toledo FiveEasy). For Ludox® TMA 1 M NaOH is also added to the particle stock up to pH 9.

The nanoparticle surface charge density σ is calculated as (Eq. 5):

$$\sigma = \frac{c * V_{eff} * q_e * N_A}{A_S * \rho * w * V} \tag{5}$$

With c the concentration of H^+ or OH^- ions added upon titration, V_{eff} the effective volume of HCl or NaOH taken up by the nanoparticles during titration, q_e the elementary charge ($1.602*10^{-19}$ C), N_A the Avogadro number ($6.022*10^{23}$ mol^{-1}), A_S the specific surface area of the nanoparticles (140 m^2/g [6]), ρ the density of the Ludox® stock dispersion (1.2 g/mL for Ludox® TMA; 1.4 g/mL for Ludox® TM50), w the nanoparticle weight fraction of the stock dispersion (0.34 for Ludox® TMA; 0.5 for Ludox® TM50) and V the volume of the nanoparticle stock dispersion used for titration.

V_{eff} represents the volume of HCl or NaOH taken up by the chargeable groups on the particle surface and not by the aqueous supernatant of the dispersion. The number of silanol groups on the particle surface is taken from adsorption isotherm measurements [6]. The volume of HCl and NaOH that affects the pH of the supernatant is subtracted from the total volume of HCl or NaOH added upon titration.

A reduction of the particle weight fraction w in the STrIPS precursor dispersion leads to a collapse of the fiber cavities (Fig. S12). This results in a loss of the cylindrical fiber shape which is accompanied by the formation of larger fiber surface pores. Fibers fabricated with a particle concentration of < 40 wt-% of the water fraction also remain fragile after UV-polymerization.

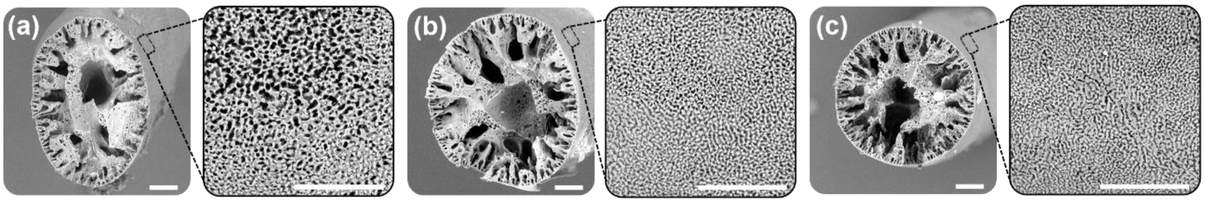

Figure S12: Scanning electron microscopy (SEM) images of cross-section and surface of STrIPS fibers (composition 3 in Fig. S2) fabricated with Ludox® TMA particle weight fractions of (a) 7.2 wt-%, (b) 8.6 wt-% and (c) 9.2 wt-% of the precursor dispersion. The CTA^+ concentration is kept at 17 mM. All scale bars 50 µm.

For increasing the particle concentration in the hollow fiber precursor (composition 2 in Fig. S2) to a total of 26 wt-% the formation of a hollow interior is inhibited (Fig. S13). Consistent with the trend in Fig. S12, the high nanoparticle concentration yields in smaller surface pores.

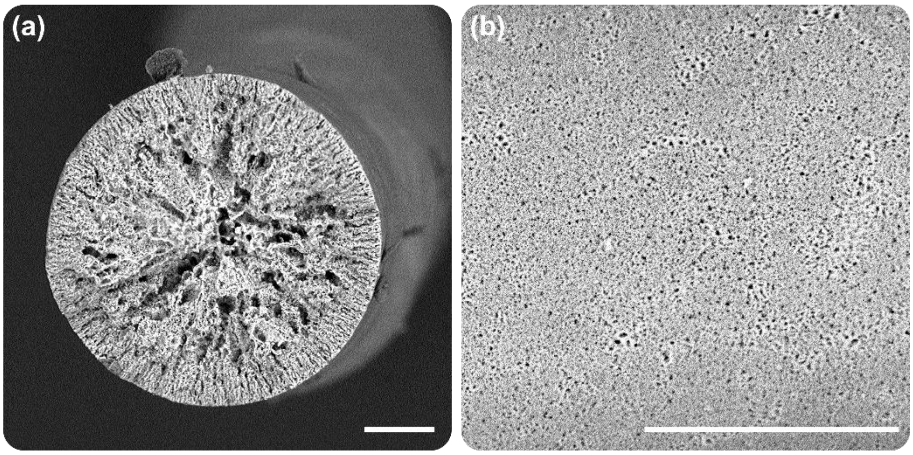

Figure S13: SEM images of (a) cross-section and (b) surface of a STrIPS fiber extruded from the hollow fiber precursor (composition 2 in Fig. S2) with a particle concentration of 26 wt-% and 17 mM CTA^+. All scale bars 50 µm.

S11. Fiber internal surface area analysis

The internal surface area of STrIPS fibers is determined from processing the confocal microscopy images of the equatorial fiber plane with the software Fiji ImageJ (version 1.53k14). The workflow is shown in Fig. S14.

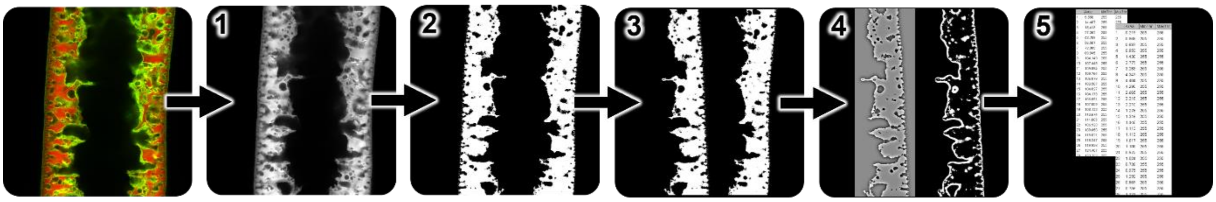

Figure S14: Measuring the STrIPS fiber surface area from confocal micrographs of the equatorial fiber plane: 1. Selecting the Nile red fluorescence of the BDA domains; 2. Enhancing contrast and binarization of the confocal microscopy image; 3. Cutting the fiber into halves in vertical direction; 4. Running a bandpass filter and skeletonization of the image to outline the interface between BDA and water; 5. Measure the number of pixels.

The white pixel skeleton in Fig. S14-4 shows the outline of the interface between BDA and water. The interfacial area can be analyzed by cutting the image of the length L into strips of ΔR = 10 pixels with radial positions R_n (Fig. S15). For each strip, the number of white pixels is measured and converted to µm with the pixel-aspect ratio of 11.0934 pixels/µm. The measured value of µm is divided by the strip area $\Delta R * L$ to obtain ϕ_n (Eq. 6). The interfacial length per strip is converted into a surface by rotation via multiplication by $2\pi R_n$ (Eq. 7). To calculate the volume-specific surface area of the fiber, the surface areas of each ring are added and divided by the total volume of the cylinder (Eq. 8).

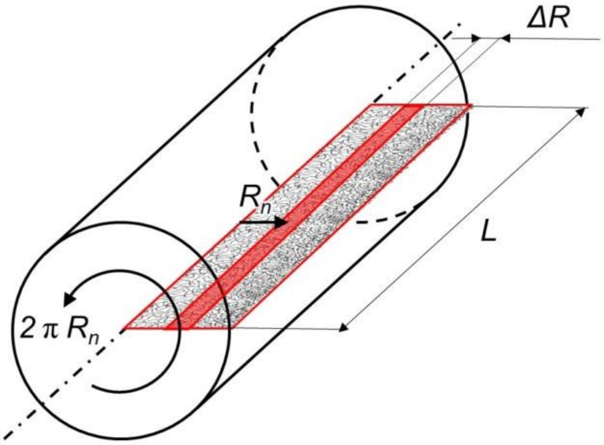

$$\phi_n = \frac{\text{white pixels per strip}}{\Delta R * L} \quad (6)$$

$$N_n = \phi_n * R_n * 2\pi * L \quad (7)$$

$$A = \frac{\sum_{n=1}^{n} N_n}{\pi * L * R^2} \quad (8)$$

Figure S15: Rotational symmetry to calculate the surface area of the cylindrical fiber segment. ΔR represents the strip width, R_n the radial position of the strip and L the strip length.

Following this procedure, we determine the internal surface area of STrIPS fibers with initial compositions 3 and 6 (Fig. S2) fabricated at different CTA^+ concentrations (Fig. S16). The confocal micrographs of these fibers are presented in Fig. 5 in the manuscript and Fig. S10 in the SI. Furthermore, the percentage of nanoparticles attached at the surface of a fiber segment, the particle attachment efficiency η, can be approximated from the fiber surface area. We calculate η by estimating the amount of particles needed to form a monolayer at the BDA/water interface. The number of particles that are required to form such a monolayer result from the area requirement of a single particle multiplied by a two-dimensional packing factor for monodisperse spheres of 0.64 (Eq. 9) [7]. This number of particles is divided by the total number of particles in the corresponding fiber segment volume (Eq. 10), giving the particle attachment efficiency η (Eq. 11).

$$N(\text{Nanoparticles at the surface}) = \frac{A}{\pi * r^2} * 0.64 \quad (9)$$

$$N(\text{Nanoparticles in fiber segment volume}) = \frac{w * \rho * V}{\rho_{NP} * 4/3 * \pi * r^3 * V_f} \quad (10)$$

$$\eta = \frac{N(\text{Nanoparticles at the surface})}{N(\text{Nanoparticles in fiber segment volume})} * 100\ \% \quad (11)$$

With N the number of nanoparticles, w the particle weight fraction and ρ the density of the Ludox® TMA dispersion, V the volume of the Ludox® TMA dispersion added to the fiber precursor dispersion, ρ_{NP} the density of the nanoparticles (2.2 g/mL), r the nanoparticle radius (10 nm) and V_f the volume of the fiber precursor dispersion.

The surface area of the STrIPS fibers increases with increasing CTA^+ concentration to a maximum of 0.24 m^2/cm^3 for BDA/ethanol/water (Fig. S16), and 0.19 m^2/cm^3 for BDA/methanol/water. This correlates with a particle attachment efficiency increasing up to 5 % for BDA/ethanol/water which is similar to 6 % for BDA/methanol/water. The low particle attachment efficiency compares to the macroporous structure of the fibers with small surface pores, while the interior is comprised of cavities.

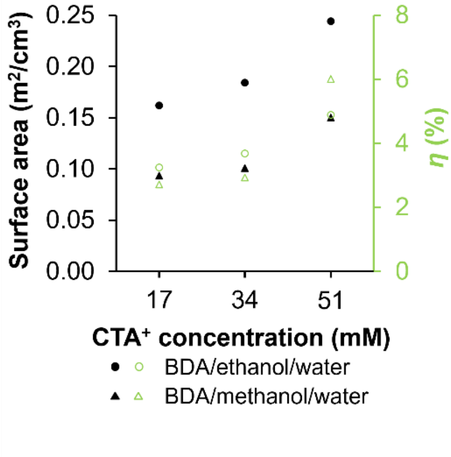

Figure S16: Surface area and particle attachment efficiency η for STrIPS fibers extruded from BDA/ethanol/water and BDA/methanol/water casting mixtures (compositions 3 and 6 of Fig. S2) using different CTA^+ concentrations. The surface area analysis corresponds to the fiber micrographs shown in Fig. 5 for BDA/ethanol/water in the manuscript and Fig. S10 for BDA/methanol/water in the SI.

S12. Surface pore size distribution of STrIPS hollow fiber membrane

The surface pore size distribution of the STrIPS hollow fiber is determined from the SEM image of the fiber surface presented in Fig. 8(a)-ii in the manuscript. Image analysis is carried out with the software Fiji ImageJ (see previous section S11): First, a bandpass filter is applied to remove image artefacts, followed by the adjustment of contrast, scale and color treshhold to expose the fiber pores. The image is binarized and inverted so that the white pixel groups accurately reflect the fiber surface pores (Fig. S17(a)). The area of the white pixel groups A is measured using the "Analyze particles" feature and converted into a mean pore diameter d with Eq. 12, assuming a circular pore shape:

$$d = \sqrt{\frac{4*A}{\pi}} \tag{12}$$

A histogram of the pore size distribution is obtained by counting the frequency of each pore size and grouping them into different size categories (Fig. S17(b)).

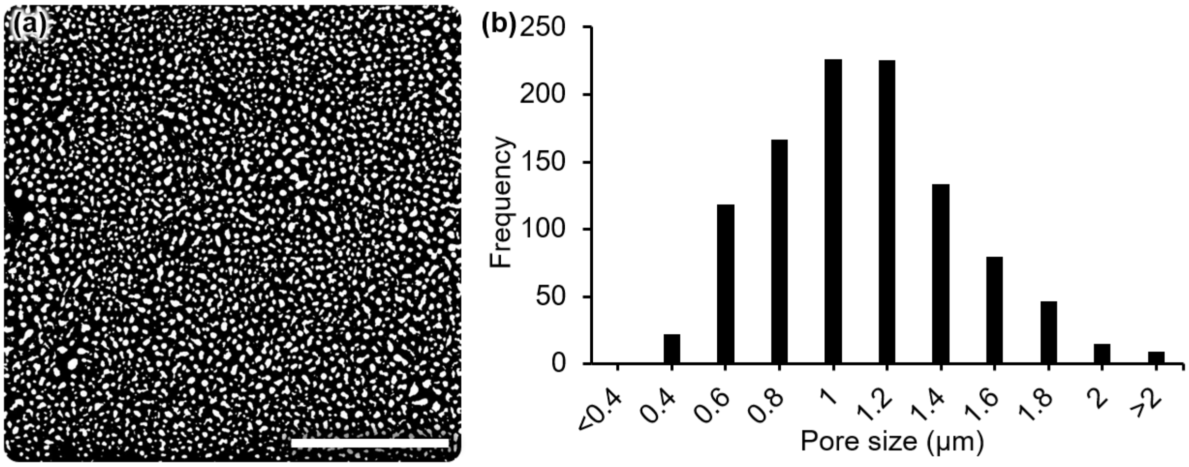

Figure S17: (a) Processed image of the STrIPS fiber surface with white dots representing the surface pores. Scale bar 25 μm. (b) Histogram of the fiber surface pore size distribution.

S13. PSS/CTA^+ complexation

To characterize the pore-closing structure that PSS and CTA^+ form, bulk precipitation experiments of aqueous solutions of PSS and CTA^+ are performed. For this experiment the molar ratio of PSS/CTA^+ is kept at 0.001 to reflect the concentrations used for the STrIPS hollow fiber coating (1 g/L PSS (= $5*10^{-6}$ mol/L) and 5 mM CTA^+; pH 5). CTA^+ is dissolved in water at concentrations ranging from 0.02-10 mM and the PSS concentration is adjusted accordingly ($2*10^{-5}$-0.01 mM). For ≥ 0.5 mM CTA^+ the samples display an increasing turbidity which can also be seen by the increasing light scattering of a laser beam shining through the samples (Fig. S18). This change in sample morphology indicates the formation of an insoluble complex of PSS and CTA^+.

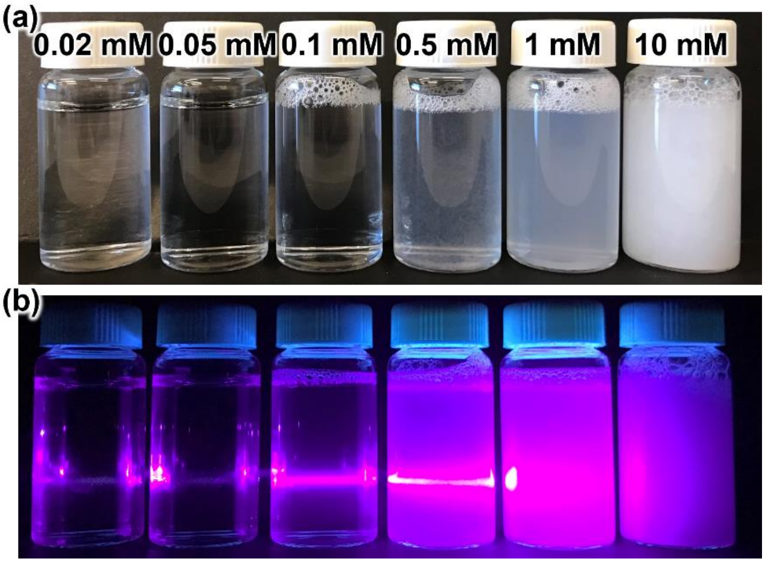

Figure S18: (a) Complexation of PSS and CTA^+. Photograph of the samples prepared with different CTA^+ concentrations (0.02-10 mM). (b) Scattering of a 405 nm laser beam shining through the samples.

S14. Polyelectrolyte functionalization of STrIPS hollow fibers

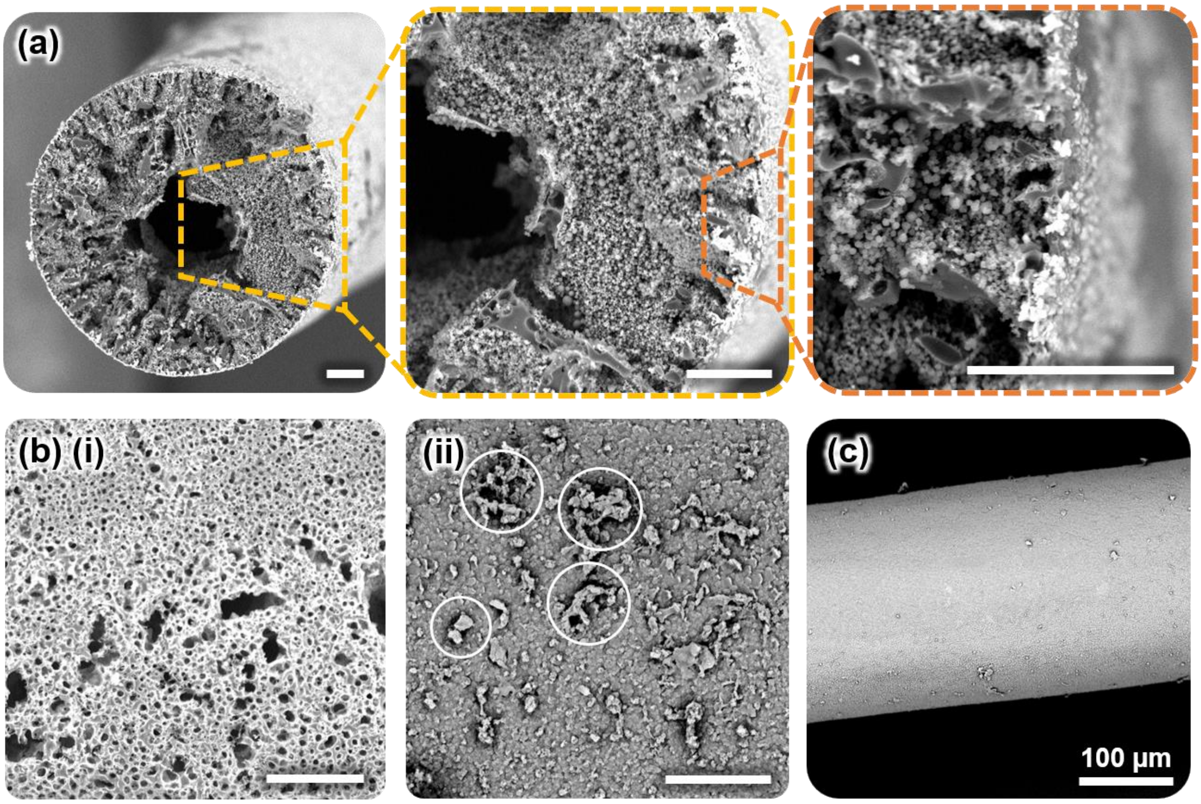

Figure S19: SEM images with zoomed-in perspectives of (a) the STrIPS hollow fiber cross-section after PSS/CTA^+ treatment to plug the fiber surfaces pores. (b) Pinhole defects at the (i) STrIPS fiber surface before polyelectrolyte modification and (ii) imperfectly clogged pinholes at the STrIPS fiber surface after coating with PSS/CTA^+-$[PDADMAC/PSS]_{50}$. (c) Overview image of a smooth, defect-free STrIPS fiber surface coated with PSS/CTA^+-$[PDADMAC/PSS]_{50}$. Scale bars 25 µm unless otherwise specified.

S15. STrIPS hollow fiber testing module

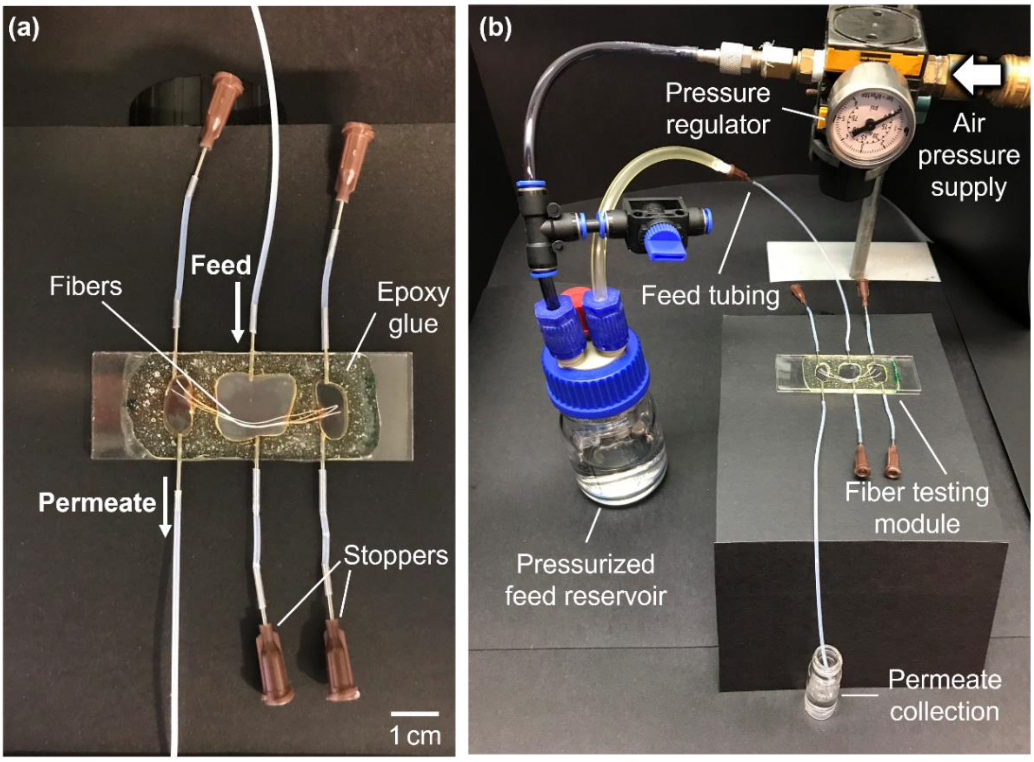

Figure S20: (a) Hollow fiber testing module. A bundle of four fibers is glued between two microscopy glass slides (Epredia) and divided into three compartments. Each compartment is connected to two cannula. The feed is supplied via the center chamber and the permeate is discharged via one of the side chambers. All other cannula are closed by stoppers. The testing module is entirely sealed with epoxy glue. (b) Experimental setup for hollow fiber flux test. The testing module is connected to a pressure-regulated feed reservoir.

The hollow fiber testing module is used for permeability testing, polyelectrolyte multilayer (PEM) coating and molecular weight cutoff (MWCO) determination. Before assembling the module, the fibers are microscopically checked for a defect-free surface (Leica M205 C). To measure hollow fiber permeability and MWCO, the testing device is connected to a pressurized water reservoir. Permeate samples are collected from the water flowing through the membrane at different pressures. For PEM coating the polyelectrolyte solutions are flown through the central chamber. After each coating step all chambers are rinsed with water to remove excess polyelectrolyte.

S16. Membrane separations with STrIPS hollow fibers

The MWCO of the STrIPS hollow fiber membranes is determined from the dextran sieving curves in Fig. S21. The MWCO is assigned to the molecular weight which is rejected to more than 90 % by the STrIPS membrane. Surface defects such as pinholes can be caused mechanically upon assembly of the fibers in the testing module (Fig. S19(b)). Likewise, STrIPS process can also entail fiber surface pores of several tens of micrometers which originate casually from fiber bending during extrusion.

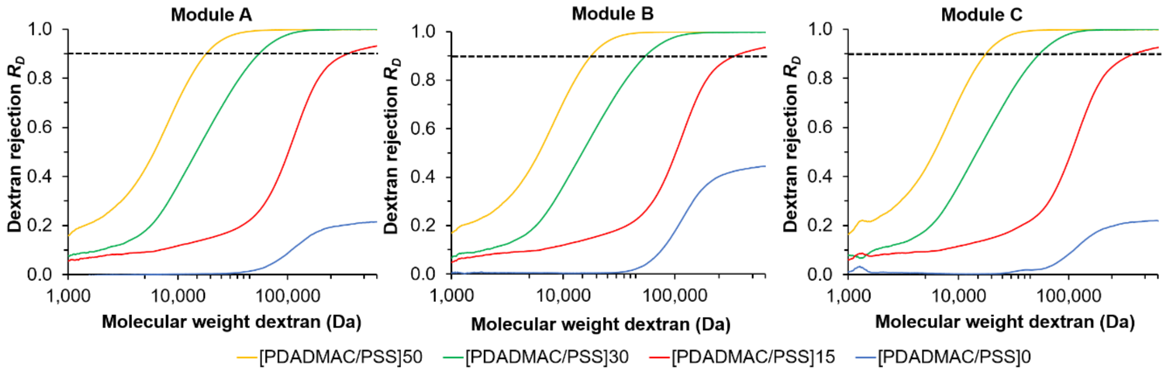

Figure S21: Sieving curves for the rejection of dextran (R_D) in dependence on the number of PDADMAC/PSS-bilayers for three different STrIPS hollow fiber testing modules A, B and C. The MWCO (defined as dextran rejection > 90 %) is indicated by the black dashed line. The PEM is built on STrIPS fibers after PSS/CTA^+ treatment. No MWCO can be detected for STrIPS fibers solely coated with a PSS/CTA^+ complex ($[PDADMAC/PSS]_0$).

Supplementary References

[1] Khan, M. A.; Sprockel, A. J.; Macmillan, K. A.; Alting, M. T.; Kharal, S. P.; Boakye-Ansah, S.; Haase, M.F. Nanostructured, Fluid-Bicontinuous Gels for Continuous-Flow Liquid–Liquid Extraction. Adv. Mater. 2022, 34, 2109547, doi: 10.1002/ADMA.202109547.

[2] Kharal, S.P.; Haase, M. F. Centrifugal Assembly of Helical Bijel Fibers for pH Responsive Composite Hydrogels. Small 2022, 18 (11), 2106826, doi: 10.1002/SMLL.202106826.

[3] Hao, L.; Leaist, D. G. Binary Mutual Diffusion Coefficients of Aqueous Alcohols. Methanol to 1-Heptanol, J. Chem. Eng. Data 1996, 41 (2), 210–213, doi: 10.1021/JE950222Q.

[4] Pratt, K. C.; Wakeham, W. A. The Mutual Diffusion Coefficient of Ethanol–Water Mixtures: Determination by a Rapid, New Method. Proc. R. Soc. A. Math. Phys. Sci. 1974, 336 (1606), 393–406, doi: 10.1098/RSPA.1974.0026.

[5] Binks, B. P.; Rodrigues, J. A.; Frith, W. J. Synergistic Interaction in Emulsions Stabilized by a Mixture of Silica Nanoparticles and Cationic Surfactant. Langmuir 2007, 23 (7), 3626–3636, doi: 10.1021/LA0634600.

[6] Atkin, R.; Craig, V. S. J.; Biggs, S. Adsorption Kinetics and Structural Arrangements of Cationic Surfactants on Silica Surfaces, Langmuir 2000, 16, 9374–9380, doi: 10.1021/la0001272.

[7] Baranau, V.; Tallarek, U. Random-Close Packing Limits for Monodisperse and Polydisperse Hard Spheres. Soft Matter 2014, 10 (21), 3826–3841, doi: 10.1039/C3SM52959B.
